# The molecular signature of heat stress in sweat reveals non-invasive biomarker candidates for health monitoring

**DOI:** 10.1038/s42003-025-08080-1

**Published:** 2025-04-23

**Authors:** Noé Brasier, Carmela Niederberger, Martina Zanella, Alaa Othman, Ralph Schlapbach, Laura Kunz, Antje Dittmann, Kelly Reeve, Michael Prummer, Jörg Goldhahn

**Affiliations:** 1 Institute of Translational Medicine, Department Health Science and Technology, ETH Zurich, Zurich, Switzerland; 2 Collegium Helveticum, Zürich, Switzerland; 3 Department of Digitalization & ICT, University Hospital Basel, Basel, Switzerland; 4 Functional Genomics Center Zurich, UZH & ETH, Zurich, Switzerland; 5 NEXUS Personalized Health Technologies, ETH Zurich, and Swiss Institute of Bioinformatics, Zurich, Switzerland

**Keywords:** Physiology, Biomarkers

## Abstract

Heat stress is a significant public health challenge that leads to an increased risk of serious health deterioration, injuries, and loss of economic productivity. While the gold standard for monitoring heat stress continues to remain with population-based measurements, a straight-forward person-centered approach is lacking. Sweat can supply a wealth of molecular information, ranging from protein levels to levels of metabolites; it is thus a promising monitoring biofluid. A thorough investigation of sweat’s molecular signature during heat stress is called for. We conducted a cross-over study on healthy participants with personalized heat-stress visits to investigate heat stress’s proteomic and molecular signatures in sweat. Through mass-spectrometry analysis, we identified multiple candidate biomarkers ranging from amino acids to microbiome metabolites and proteins. To the best of our knowledge, these biomarker candidates represent the first successful approach to metabolically differentiate between various heat stressors thereby enabling their acute monitoring. While these biomarker candidates need further investigation to confirm their clinical value, many have already been identified as directly associated with heat stress in animals and plants. Once further investigated, next-generation wearable devices for person-centered, on-skin sweat-analysing platforms could be developed that would transform health management during exposure to heat stress.

## Abstract (web summary)

The molecular signature of heat stress in sweat reveals promising biomarker candidates for heat stress monitoring and detection. Once validated, these biomarkers can be clinically implemented through sweat analysis by wearable devices.

## Body

Climate change is one of the major public health challenges of the 21^st^ century^1,2^. Due to climate change, most countries will be exposed to heat stress by the end of the 21^st^ century^3,4^. Heat stress can lead to significant health issues ranging from dehydration to heat stroke, with a consequent increased risk of death^5^, and heat stroke can even occur at lower temperatures^6^. Important, about 19% of exertional heat stroke appeared in a non-hot environment ( < 15 °C) a study found^7^. Overall, heat stress affects health, lowers our work performance by up to 40%, and increases the risk of work-related injuries^8,9^.

Heat stress leads to a variety of physiological reactions—known as heat strain—to maintain thermoregulatory homeostasis at an ideal core body temperature (CBT) of around 37 °C^10^. The heat-strain involves changes in basic vital parameters related to hemodynamic and the sudomotor functions^11,12^. In this context, whole-body sweat loss is an indicator of the heat that must be dissipated to maintain thermoregulatory homeostasis^13^. Various factors, such as age, physical fitness, and thermal acclimatization modulate the heat-strain reaction^14–17^. An individual’s heat acclimatization status can change, for example due to (i) travelling to different thermal zones for an extended period of time, (ii) being constantly exposed to air conditioning, and (iii) wearing different types of clothing^18,19^. Heat-stress and heat-strain monitoring, including interpretation for health management purposes, is challenging. While the gold standard for monitoring heat stress is based on population-based assessments, a personalized approach is thoroughly lacking. Wearable devices allow for personalized heat-strain measurements by enabling continuous and lab-independent health monitoring but often rely on nonspecific physical variables such as heart rate (HR).

Novel wearable devices have been introduced that can monitor electrolytes, glucose, proteins, and metabolites excreted in sweat^20,21^. Despite the advances in remote hydration monitoring through on-skin sweat analysis for optimizing health management during heat exposure^22^, heat-strain monitoring has been predominantly based on physical assessments; a metabolic monitoring approach still needs to be devised. Importantly, even though physical heat-strain measurements are assessable through wearable sensors, their interpretation can be challenging. For example, CBT measurements can surpass the broadly accepted safety limit of 39 °C without necessarily leading to significant physiological impairment^23^. Several molecular heat-strain biomarkers such as cortisol and α-amylase in blood, are associated with heat stress^24,25^. Their standard detection and monitoring are neither continuous nor possible outside of laboratories. Non-invasive and continuous biochemical heat-strain monitoring through body-fluid analysis by wearable devices therefore harbors great potential^26^.

Human sweat contains a wealth of molecular information, ranging from proteins to metabolites, and drugs^27^. The rate and molecular composition of sweat vary with a person’s physical-environmental exposure but also depend on the collection method—a fact which must be given careful consideration^28^. Sweat is non-invasively and continuously collectable from the skin surface. The current gold-standard for assessing the molecular composition of sweat continues to involve extensive classical lab analytics such as mass spectrometry. However, emerging wearable devices for sweat analysis have already demonstrated their potential to straightforwardly and biochemically monitor amino acids, lactate, cortisol, steroid hormones, and inflammation markers^29–33^. However, to the best of our knowledge, a comprehensive proteomic and metabolomic exploration of sweat’s biochemical composition to determine extended metabolic biomarkers for heat-strain monitoring is still lacking.

In this exploratory study, we investigated the metabolomic and proteomic molecular signature of heat stress in the sweat of healthy participants during exposure to various heat stressors. In a personalized study setting, participants were exposed to various heat stressors: (i) elevated ambient temperature (AT), (ii) elevated relative humidity (RH), (iii) exertion (E), and (iv) protective clothing (PC). Sweat was collected using commercially available medical patches and molecular analysis was carried out via mass spectrometry. The study aimed at identifying novel molecular biomarker candidates in sweat that will enable the improvement of state-of-the-art heat-stress monitoring in the future. Identifying these biomarkers provides a first step towards better understanding the underpinning human metabolic mechanisms associated with heat strain. This exploration could help to develop a tool for better illuminating and managing the mechanisms of heat-strain.

## Results

### Participant characteristics

**Fig. 1:**
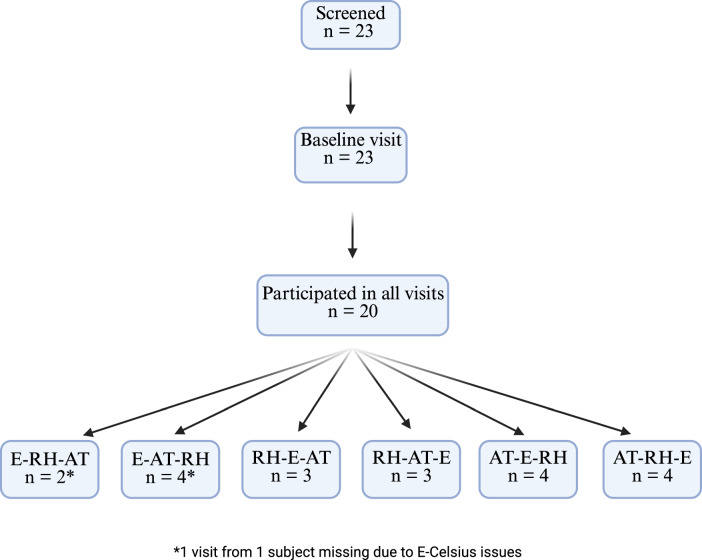
Flow of study participants. E indicates exertion, RH indicates relative humidity, and AT indicates ambient temperature. All 20 participants were exposed to the three conditions E, RH, and AT. To minimize the potential influence of visit order, the sequence of these three conditions was randomized. This approach, however, resulted in small sample sizes for specific visit orders.

A total of 23 participants were recruited between November 2022 and February 2023 (Fig. 1). Three participants chose to drop out. Due to sensor failure, two study visits had to be terminated early and thus were excluded from analysis. Ten female and ten male participants were included in the analysis. The mean close body temperature (T_CB_)—the temperature measured about 6 mm above the body surface using an iButton sensor—at home was 29.89 °C (SD ± 1.89) and the mean close body relative humidity (RH_CB_) was 37.08% (SD ± 7.47). Participants’ baseline characteristics are displayed in Table 1.

**Table. 1:** Baseline characteristics of the study participants

	Mean (sd)
Number of participants (*n*)	20
Age (years)	24.60 (4.37)
Male (%)	10 (50.0)
Height (m)	1.75 (10.20)
Weight (kg)	66.88 (10.98)
BMI (kg/m^2^)	21.80 (2.28)
BSA (m^2^)	1.81 (0.19)
VO2 max (ml/(kg*min)	46.62 (6.93)
Max HR (n/min)	166.75 (3.29)
Close body temperature at home (°C)	29.89 (1.89)
Close body relative humidity at home (%)	37.08 (7.47)

The estimated whole-body sweat loss per body surface area was the highest when the participants were exposed to E (mean 0.3 L/m^2^ ± 0.07 L/m^2^), medium when they were exposed to AT (0.16 L/m^2^ ± 0.05 L/m^2^), and lowest when they were exposed to RH (mean 0.07 L/m^2^ ± 0.03 L/m^2^) (Supplemental Material 1). In general, local sweat rates were highest during E and the lowest during RH stress (Supplemental Material 1). The Wet-bulb globe temperature (WBGT), representing environmental heat stress, showed significant differences during AT (incl. PC) and RH (incl. PC), compared to E, where the focus was on active heat stress (Supplemental Material 2).

### Untargeted metabolomic and proteomic signatures in sweat

#### Metabolomics

An untargeted metabolomic analysis of sweat identified a total of 82 unique metabolites, including 31 endogenous and 51 exogeneous markers such as extractables and additives. During the stress phase (without PC), metabolite intensities appeared to be higher for most participants during AT and E than during RH. However, not all metabolites showed a significant change with the various phases.

#### Proteomics

137 proteins were observed in at least 50% of samples. Proteins were less likely to be detected during low-sweat-rate conditions, such as the RH stress phases and the acclimation and cool-down phases.

### Differentiation between heat stressors

#### Metabolomic

The intensities of four endogenous metabolites in sweat were identified for which there was at least moderate evidence (even after multiplicity correction) of differences in intensity between the heat stressors AT, RH, E, and +PC (Table 2A). The identified metabolites were *trans-3-Indoleacrylic acid*, *cis,cis-Muconic acid*, *leu-leu* (dipeptide based on the amino acid *leucine*), and *leu-phe* (dipeptide based on the amino acids *leucine* and *phenylalanine*).

**Table 2:** A-B. Top 10 metabolomic (A) and proteomic (B) biomarker candidates in sweat and differentiation between the various heat stressors. Metabolite results are shown when significant under both the standard and addition clothing stresses

Analytics	Name	contrast	Estimate	SE	t	pvalue	p_adjusted	Percentage Missing (%)
A) Metabolomics	trans-3-Indoleacrylic acid	Ambient Temperature - Relative Humidity	1.856	0.3775	4.9166	<0.0001	<0.0001	0
	Leu-Phe	Ambient Temperature - Relative Humidity	1.9297	0.4225	4.5679	<0.0001	<0.0001	0
	trans-3-Indoleacrylic acid	Relative Humidity - Exertion	−2.9482	0.3775	−7.8099	<0.0001	<0.0001	0
	Leu-Phe	Relative Humidity - Exertion	−3.0083	0.4225	−7.1209	<0.0001	<0.0001	0
	Leu-Leu	Relative Humidity - Exertion	−2.2488	0.3766	−5.9717	<0.0001	<0.0001	0
	cis,cis-Muconic acid	Relative Humidity - Exertion	0.9586	0.193	4.9667	<0.0001	<0.0001	0
	Leu-Leu	Ambient Temperature - Relative Humidity	1.2916	0.3766	3.4298	0.0008	0.002	0
	trans-3-Indoleacrylic acid	Ambient Temperature - Exertion	−1.0922	0.3823	−2.8569	0.005	0.012	0
	cis,cis-Muconic acid	Ambient Temperature - Relative Humidity	−0.4857	0.193	−2.5164	0.013	0.027	0
	Leu-Phe	Ambient Temperature - Exertion	−1.0786	0.4278	−2.5209	0.013	0.027	0
B) Proteomics	Fibromodulin	Relative Humidity - Exertion	−5.6023	1.0298	−5.4402	<0.0001	<0.0001	35.8
	Fibromodulin	Ambient Temperature - Relative Humidity	3.4002	1.0298	3.3018	0.001	0.005	35.8
	Fibromodulin	Ambient Temperature - Exertion	−2.2021	1.0429	−2.1115	0.036	0.078	35.8

AT indicates ambient temperature, RH indicates relative humidity, E indicates exertion.

#### Proteomic

In the screening process, with intensity trajectories significantly different for all three stressors (AT, RH, E) and +PC, no proteins were identified as potential biomarkers. However, when focusing on AT, RH, E without +PC, there was at least moderate evidence of differences in intensities across heat stressors for *fibromodulin* (Table 2B), although the T vs. E evidence weakened at multiplicity correction.

### Differentiation between low and high WBGT

#### Metabolomic

Comparing the metabolite intensities in sweat between heat-stress environments with low (mean

27.74 °C; RH) and high (mean 31.26 °C; AT) WBGT led to the identification of 25 biomarker

candidates (Table 3a). The biomarker candidates ranged from amino acids

**Table 3:** A-B. Top 10 metabolomic (A) and proteomic (B) biomarker candidates for distinguishing between high Wet-Bulb Globe Temperature (WBGT) (ambient temperature visit) and low Wet-Bulb Globe Temperature (relative humidity visit) intensities of ambient heat stress

Analytics	Name	Contrast	Estimate	SE	t	pvalue	p_adjusted	Common (0,1)	Percentage missing (%)
A) Metabolomics	Urocanic acid	High WBGT - Low WBGT	3.392088	0.554183	6.120875	6.56E-09	9.72014E-08	1	0
	Phenylalanine	High WBGT - Low WBGT	2.857268	0.483156	5.913765	1.87E-08	2.32782E-07	1	0
	Tryptophan	High WBGT - Low WBGT	3.692822	0.624899	5.909468	1.91E-08	2.32782E-07	1	0
	Norleucine	High WBGT - Low WBGT	2.696545	0.478436	5.636161	7.37E-08	7.61483E-07	1	0
	Histidine	High WBGT - Low WBGT	3.432838	0.624758	5.494666	1.46E-07	1.34461E-06	1	0
	Citrulline	High WBGT - Low WBGT	1.957507	0.382019	5.124106	8.28E-07	5.7981E-06	1	0
	trans-3-Indoleacrylic acid	High WBGT - Low WBGT	1.855984	0.377495	4.916579	2.11E-06	1.33358E-05	1	0
	Serine	High WBGT - Low WBGT	2.031319	0.418321	4.855887	2.76E-06	1.68296E-05	1	0
	Leu-Phe	High WBGT - Low WBGT	1.929734	0.42246	4.567853	9.6E-06	5.28171E-05	1	0
	Arginine	High WBGT - Low WBGT	3.173618	0.700977	4.527421	1.14E-05	6.16471E-05	1	0
B) Proteomics	Glutaminyl-peptide cyclotransferase	High WBGT - Low WBGT	5.342356	0.799517	6.681977	3.46E-10	1.04158E-07	1	47.8
	Lysosomal Pro-X carboxypeptidase	High WBGT - Low WBGT	5.407522	0.816884	6.619697	4.82E-10	1.21175E-07	1	40.1
	Isocitrate dehydrogenase [NADP] cytoplasmic	High WBGT - Low WBGT	4.623558	0.723624	6.389452	1.63E-09	2.56733E-07	1	45.3
	N(4)-(beta-N-acetylglucosaminyl)-L-asparaginase	High WBGT - Low WBGT	5.231822	0.866247	6.039639	9.92E-09	9.34009E-07	1	40.1
	Carboxypeptidase M	High WBGT - Low WBGT	4.542361	0.765512	5.933754	1.69E-08	1.34221E-06	1	49.6
	Glutathione synthetase	High WBGT - Low WBGT	4.419596	0.755702	5.84833	2.59E-08	1.72244E-06	1	48.7
	Proteasome subunit alpha type-1	High WBGT - Low WBGT	4.778947	0.8391	5.695324	5.52E-08	2.97214E-06	1	43.1
	Serpin B6	High WBGT - Low WBGT	4.541565	0.797427	5.695271	5.52E-08	2.97214E-06	1	46.1
	Catalase	High WBGT - Low WBGT	5.446057	0.961607	5.663494	6.45E-08	3.2402E-06	1	33.2
	Ester hydrolase C11orf54	High WBGT - Low WBGT	4.969556	0.900143	5.520852	1.29E-07	5.05013E-06	1	47.4

For *Common*, 0 indicates that p-value accounts for either normal clothing or protective clothing, 1 indicates that the p-value accounts for both normal and +PC.

(*histidine*, *tryptophan*, *citrulline*, *phenylalanine*, and *norleucine*) to human microbiome-related markers (*trans-3-Indoleacrylic acid* and *cis,cis-Muconic acid*). While most biomarker candidates enabled differentiation between low and high WBGT within the heat stress visit, statistically significant changes between the heat stress and +PC phase were only found for *pipecolic acid*,

#### N-(2-hydroxyphenyl) acetamide, 9-Oxo-10(E),12(E) octadecadienoic acid, palmitoyl

*ethanolamide*, *2-Amino-1,3,4 octadecanetriol*. Most evidence was still at least moderately strongafter multiplicity correction.

#### Proteomic

A total of 128 proteins allowing for a statistically significant differentiation between high and low WGBT were identified in sweat (Table 3b). While most proteins enabled differentiation between high and low heat stressors during both the single stress and +PC phases, statistically significant differences between high and low heat stressors were only found for either the single stress or +PC phase for the following proteins: *melanotransferrin*, *desmoplakin*, *trypsin-3*, *keratin*, *type I cytoskeletal 9*, *origin recognition complex subunit 1*, *filaggrin-2*, *dermokine*, *nesprin-1*, *mucin-like protein 1*, and *mitochondrial ribosome-associated GTPase 1*. However, the missing data for these potential biomarkers was high, with a maximum of 49.6% (median 37.5%). Most evidence was still at least moderately strong after multiplicity correction.

### Differentiation between ambient and active heat stress

#### Metabolomic

By comparing the molecular signatures in sweat during heat-stress phases from environmental sources (RH, AT) and being active (E) under conditions without ambient heat stress, 26 biomarker candidates were identified (Table 4A). The biomarker candidates include amino acids (*histidine*, *tryptophan*, *citrulline*, *phenylalanine*, and *norleucine*), human microbiome–related markers (*trans-3-Indoleacrylic acid* and *cis,cis-Muconic acid*) and the steroid hormone *testosterone*. While most candidates enabled differentiation during both the single heat stress and +PC phases, *norleucine*, *citrulline*, *testosterone*, and *9-oxo-10(E)*,*12(E)-octadecadienoic acid* did not. Only weak evidence was found for differences in trajectories were found such as for *pipecolic acid*, *perillartine*, *hexadecanamide*, *sphingosine*, *stearoyl ethanolamide* after correction, while evidence remained at least moderately strong for all other relationships.

**Table 4:** A-B. Top 10 metabolomic (A) and proteomic (B) biomarker candidates for differentiating between environmental and active heat stressors

Analytics	Name	Contrast	Estimate	SE	t	p-value	p_adjusted	Common	Percentage missing (%)
A) Metabolomics	trans-3-Indoleacrylic acid	Environmental Heat Stress - Active Heat Stress	-2.02019	0.329703	-6.12731	6.34E-09	9.72014E-08	1	0
	Tryptophan	Environmental Heat Stress - Active Heat Stress	-3.21146	0.545784	-5.88412	2.17E-08	2.55137E-07	1	0
	Leu-Phe	Environmental Heat Stress - Actve Heat Stress	-2.04342	0.368975	-5.53811	1.18E-07	1.12184E-06	1	0
	Urocanic acid	Environmental Heat Stress - Active Heat Stress	-2.62192	0.484022	-5.41695	2.11E-07	1.7612E-06	1	0
	Phenylalanine	Environmental Heat Stress - Active Heat Stress	-2.13808	0.421986	-5.06672	1.08E-06	7.33164E-06	1	0
	Leu-Leu	Environmental Heat Stress - Active Heat Stress	-1.60299	0.328897	-4.87385	2.55E-06	1.58276E-05	1	0
	Norleucine	Environmental Heat Stress - Active Heat Stress	-1.87636	0.417864	-4.49035	1.33E-05	6.98017E-05	0	0
	Histidine	Environmental Heat Stress - Active Heat Stress	-2.36373	0.545661	-4.33186	2.56E-05	0.000126526	1	0
	cis,cis-Muconic acid	Environmental Heat Stress - Active Heat Stress	0.715771	0.168571	4.246103	3.62E-05	0.000171584	1	0
	Arginine	Environmental Heat Stress - Active Heat Stress	-2.44137	0.61223	-3.98767	0.0001	0.000431897	1	0
B) Proteomics	Glutaminyl-peptide cyclotransferase	Environmental Heat Stress - Active Heat Stress	-3.25227	0.698295	-4.65744	6.56E-06	7.37271E-05	1	47.8
	Isocitrate dehydrogenase [NADP] cytoplasmic	Environmental Heat Stress - Active Heat Stress	-2.92168	0.63201	-4.62284	7.6E-06	8.10733E-05	1	45.3
	Lymphocyte antigen 6D	Environmental Heat Stress - Active Heat Stress	-3.02868	0.663149	-4.56712	9.63E-06	9.67798E-05	1	49.1
	Serpin B6	Environmental Heat Stress - Active Heat Stress	-3.07734	0.69647	-4.41848	1.79E-05	0.000156832	1	46.1
	Fibromodulin	Environmental Heat Stress - Active Heat Stress	-3.90221	0.899421	-4.33858	2.49E-05	0.000196539	1	35.8
	Lysosomal Pro-X carboxypeptidase	Environmental Heat Stress - Active Heat Stress	-3.0511	0.713463	-4.27646	3.21E-05	0.000234515	1	40.1
	Pro-cathepsin H	Environmental Heat Stress - Active Heat Stress	-3.10742	0.7704	-4.03352	8.38E-05	0.000546948	1	39.7
	Aspartate aminotransferase, cytoplasmic	Environmental Heat Stress - Active Heat Stress	-3.0337	0.759621	-3.99371	9.78E-05	0.000619022	1	42.2
	Glutathione synthetase	Environmental Heat Stress - Active Heat Stress	-2.61252	0.660027	-3.9582	0.000112	0.000700446	1	48.7
	N(4)-(beta-N-acetylglucosaminyl)-L-asparaginase	Environmental Heat Stress - Active Heat Stress	-2.98032	0.756577	-3.93922	0.00012	0.000731785	1	40.1

For *Common*, 0 indicates that the p-value accounts for either normal clothing or protective clothing, 1 indicates that the p-value accounts for both normal and +PC.

#### Proteomic

A total of 89 proteins were identified that allowed differentiation between environmental (AT and RH) and active (E) heat stress (Table 4B). Again, there was a high amount of missing data (up to 49.6%; median 41.8%).

### Association with physical heat-strain markers

#### Metabolomics

Significant correlations were found between biomarker candidates in sweat and CBT and HR. Only *citrulline* demonstrated a significant correlation with CBT for all visits (Table 5A).

**Table 5:** A-B. Correlations between metabolomic (A) and proteomic (B) biomarker candidates and core body temperature (CBT) and heart rate (HR)

Vital Parameter	Analytics	Name	Estimate	SE	t	pvalue	p_adjusted
A) Heartrate	Metabolomics	Sphingosine	-0.0049	0.0023	-2.1153	0.036	0.29
		6-Hydroxynicotinic acid	0.0067	0.0032	2.0915	0.038	0.29
	Proteomics	Filaggrin-2	-0.0237	0.0104	-2.2772	0.024	1.00
		Repetin	-0.0226	0.0108	-2.0924	0.038	1.00
		Zinc finger MYM-type protein 3	0.02	0.0097	2.062	0.04	1.00
B) Core body temperature	Metabolomics	Citrulline	-0.3758	0.1736	-2.165	0.031	0.77

There was evidence of a correlation between HR and *6-hydroxynicotinic acid* and *sphingosine* (Table 5B). However, no evidence of relationships between metabolites and the vital parameters was found after multiplicity correction.

#### Proteomics

Correlation of the proteomic analysis with monitored vital parameters was assessed. No correlation was found between CBT and proteins in sweat. HR correlated with three proteins, namely *fillaggrin-2, repetin*, and *zinc finger MYM-type protein 3* (Table 5A). Again, however, no evidence was available after correction.

### Monitoring acute heat stress

#### Metabolomics

**Fig. 2:**
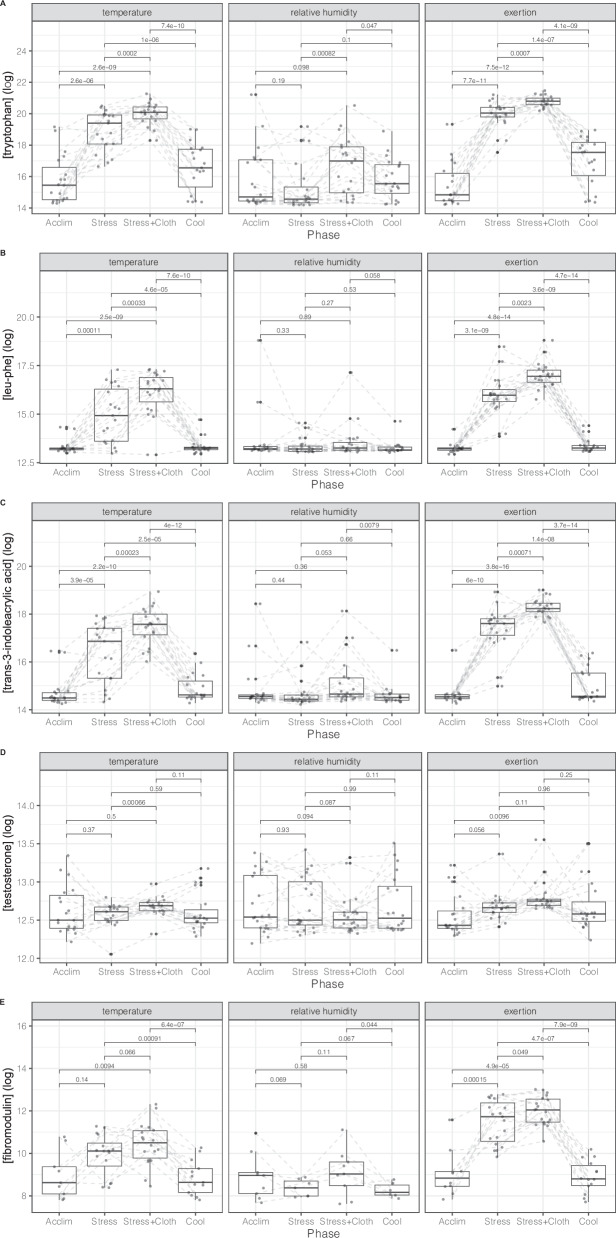
Monitoring acute heat stress through metabolomic sweat analysis. Changes of intensities of (**A**) *tryptophan*, (**B**) *leu-phe*, (**C**) *trans-3-indoleacrylic acid*, (**D**) *testosterone*, and (**E**) *fibromodulin* intensities in sweat during exposure to the heat stressors along the study visits. The boxplots are drawn according to a standard definition: Boxes indicate the range between the first and third quartile, whiskers are 1.5 times the inter quartile range and up to the last data point at maximum.

Multiple biomarker candidates for heat-strain monitoring were identified, correlating with changes of heat stress during the respective visits, especially under conditions of E and AT. The focus in this first exploratory analysis was to identify novel metabolomic biomarker candidates for participants’ heat strain. *Tryptophan* enabled the monitoring of heat-stress changes between all study phases with a high statistical significance in E and AT, changes from heat stress to heat stress +PC, and a subsequent change in the cool-down phase in RH (Fig. 2A). For *leu-phe*, changes in heat stress were monitorable during E and AT but not RH (Fig. 2B). Changes of *trans-3-indoleacrylic acid* intensities in sweat enabled differentiation between the baseline and the different heat stress phases including the recovery phase with only one exception for RH (Fig. 2C). Finally, *testosterone* intensities in sweat enabled differentiation between (i) heat stress and heat stress +PC during AT and (ii) the acclimatization and heat stress plus clothing phase in E (Fig. 2D).

#### Proteomics

*Fibromodulin* allowed the monitoring of heat-stress changes during the various visit phases, best during E but also partly during AT and RH (*p *< 0.05 – *P* < 0.0001; Fig. 2E). Interestingly, *fibromodulin* intensities in sweat enabled the monitoring of recovery during the transition from heat stress +PC to cool-down for all three heat stressor visits (E, AT, RH). However, many data points were missing, especially during the RH phase.

### Targeted cortisol signature in sweat

As *cortisol* in blood is a well-established marker of both general and heat stress, a targeted, absolute quantification of *cortisol* was conducted. In sweat, *cortisol* was detectable during all phases including the acclimatization phase with very low sweat rates, even though its detection was low in low-stress tasks. Compared to RH, *cortisol* was significantly more likely to be detectable in phases of higher heat stress induced through AT or E. However, no evidence of a difference in *cortisol* detectability between AT and E was found, even though there was a trend to a higher likelihood of *cortisol* detection in E than in AT (Table 6A).

**Table 6:** A-B. The detectability of *cortisol* in sweat during heat-stressor visits (A)

A) Detectability of Cortisol in Sweat		
Heatstress type	Phase	Mean detectability in % (Range)
Ambient Temperature	Acclimatization	10.5 (2.9 to 31.4)
Ambient Temperature	Stress	63.2 (41 to 80.9)
Ambient Temperature	Stress+ Protective Clothing	84.2 (62.4 to 94.5)
Ambient Temperature	Cool	26.3 (11.8 to 48.8)
Relative Humidity	Acclimatization	15 (5.2 to 36)
Relative Humidity	Stress	15 (5.2 to 36)
Relative Humidity	Stress+ Protective Clothing	5 (0.3 to 23.6)
Relative Humidity	Cool	10 (2.8 to 30.1)
Exertion	Acclimatization	15.8 (5.5 to 37.6)
Exertion	Stress	100 (83.2 to 100)
Exertion	Stress+ Protective Clothing	100 (83.2 to 100)
Exertion	Cool	21.1 (8.5 to 43.3)

B) Association of Cortisol with vital parameters						
Univariable	Multivariable					
	Coefficient	95% confidence interval	pvalue	Coefficient	95% confidence interval	pvalue
Intercept0	26.70	from 3.64 to 49.77	0.025*	17.22	from -10.41 to 44.85	0.20
Mean CBT	-0.52	from -1.14 to 0.10	0.096	-0.22	from -1.01 to 0.57	0.56
Intercept1	8.65	from 7.37 to 9.92	< 0.0001*			
Mean HR	-0.013	from -0.02 to -0.00	0.031*	0.0088	from -0.01 to 0.03	0.28
Intercept2	9.70	from 8.38 to 11.03	< 0.0001*			
Sweat rate (log)	-0.45	from -0.69 to -0.21	0.0006*	-0.40	from -0.95 to 0.15	0.14
Intercept3	7.87	from 7.10 to 8.65	< 0.0001*			
Heat shock protein (log)	-0.24	from -0.53 to 0.05	0.10	-0.21	from -0.50 to 0.07	0.13

The association between cortisol, vital parameters, and heat-shock proteins (B).

**Fig. 3:**
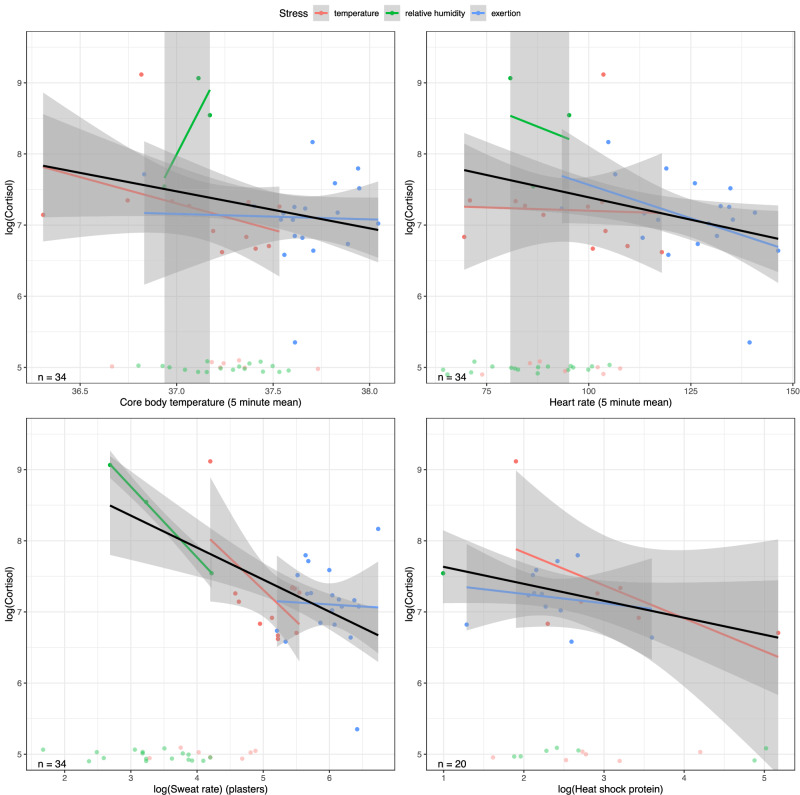
Targeted cortisol signature in sweat. Scatter plots of log cortisol concentrations versus each of the physical parameters core body temperature, heart rate, sweat rate, and heat shock protein. In addition, full lines show the least square linear regression results, where the black line corresponds to a model over all stressors. The grey areas represent the pointwise 95% confidence intervals.

*Cortisol* was more often detected in the stress and stress +PC phases than in the acclimatization and cool-down phases. Univariate analysis demonstrated an inverse relationship between *cortisol* concentrations, HR, and LSR, but no such relationship with CBT or heat-shock protein intensities (Table 6B; Fig. 3). This result fits with earlier investigations demonstrating a reduction of plasma cortisol in low-intensity exercise^34^ in which HR and LSR increase due to stress, but heat stress was not significant enough to clearly raise CBT. However, these results did not withstand multivariate analysis.

## Discussion

This personalized, observational study explored the metabolomic and proteomic signatures in sweat during various heat stress conditions such as AT, RH, E, and clothing PC with an eye towards the development of non-invasive and continuous molecular heat-strain monitoring. Untargeted mass spectrometry provided a signature of 31 endogenous metabolite and 137 protein biomarker candidates in addition to *cortisol* in sweat. Several of these molecular candidates allowed the monitoring of and differentiation between acute heat stress changes and stressors. The metabolomic markers were easily detectable even under low-sweat-rate conditions, while such conditions resulted in a high percentage of missing data for the proteomic analysis. Only a few of the identified molecules correlated significantly with established physical heat-strain measurements. Thus, the assessment of the molecular signature of sweat may add important information to that obtained through mere physical monitoring. The ability to (i) identify the metabolic impact of heat stress even at moderate WBGT levels and (ii) differentiate between environmental and active heat stress harbors significant potential: it could expand on the limited heat-strain information currently derived through physical measures and ultimately help tailor and refine health management in conditions of elevated heat. The biomarker candidates identified in our study could be straightforwardly integrated into recently developed wearable sensing platforms for on-skin sweat analysis^22,30,31,35^, thereby enabling translation into extended continuous, non-invasive, and lab-independent active heat-strain sensing.

Amino acids (AA) play an important role in the response to heat stress across species, among them many plants, fungi, and vertebrates—for example, in broiler chickens, cows, and humans^36–41^. Most studies investigating the association between AA concentrations and heat stress have been conducted in non-humans; data on AAs as biomarkers for heat-strain assessment in humans thus remains scarce.

In chickens, plasma concentrations of AAs such as *tryptophan* were demonstrably affected by acute and chronic heat stress^36,37^, increasing during acute heat-stress exposure (15–30 min) and decreasing during longer periods of heat stress exposure (24–48 h). Studies in plants and broilers have underlined *tryptophan*’s potential as a marker of heat strain^38,42^. In broilers, *tryptophan* supplementation has even led to heat-strain relief^42,43^. In humans, *tryptophan* has a key function in the synthesis of 5-hydroytryptamine (5-HT; Serotonin)^44^, which is involved in heat strain during exercise^45,46^. Further, pathological changes in various diseases of the respiratory, cardiovascular, and nervous systems are associated with a change in *tryptophan* metabolism^47,48^. These comorbidities are known to increase heat susceptibility^5,49,50^. Additionally, in the late phase of acute heat stroke, epileptic seizures may occur, and seizures themselves have been associated with changes in plasma *tryptophan* concentrations^48^. Finally, *tryptophan* has an important role in maintaining cognitive function and stabilizing mood^51^, both of which can be strongly affected during heat exposure, with serious occupational health implications for safety and productivity. In this study, *tryptophan* intensities in sweat allowed (i) discrimination between low and high heat stress as measured by WBGT, (ii) discrimination between ambient and active heat stress, and (iii) the monitoring of heat-stress changes across different visit phases.

*Trans-3-Indoleacrylic acid* was identified as another biomarker candidate allowing differentiation between all the heat stressors and most of the visit phases. Interestingly, *trans-3-Indoleacrylic acid* is a compound derived from the metabolization of *tryptophan* through the human microbiome, which in turn contributes to the stabilization of the human metabolism^52^. Therefore, *tryptophan* and its microbiome derivative in sweat—*trans-3-Indoleacrylic acid*—are promising biomarker candidates for acute-heat-strain monitoring and for distinguishing between heat stressors. Further studies in humans are needed to investigate underpinning causalities.

The dipeptides *leu-leu* (dipeptide made of two *leucine* AAs) and *leu-phe* (dipeptide made of the AAs *leucine* and *phenylalanine*) allowed significant differentiation between all the heat stressors and between the visit phases. In broilers, *leucine* injected in ovo has been shown to impact thermotolerance during heat stress after hatching^53^. Interestingly, females showed a greater reduction of rectal temperature than males. *Phenylalanine* is an essential AA with a significant role in the synthesis of *tyrosine* and ultimately *catecholamines*. *Catecholamines* are important in the physiological heat-strain reaction; for example, they increase cardiovascular function by changing heart rate and blood pressure^54^. Studies in chickens have shown that short periods of heat stress had no effect on plasma *phenylalanine* levels but that prolonged heat exposure was associated with a decrease of *phenylalanine* in plasma^55^. In rodents, *phenylalanine* improves glucose tolerance and suppresses food intake, both common physiological reactions to heat stress^56,57^. The AA *citrulline* is demonstrably involved in heat-shock response and in the central regulation of CBT in broilers^58^. In animals, *citrulline* supplementation has been associated with the relief of heat-stress symptoms^59^. In our study, *citrulline* was the only metabolomic marker in sweat correlating negatively with the CBT of participants. Thus, *citrulline* may help to better understand the interpretation of CBT in the context of heat-stress exposure and potentially infection related fever.

In mice, elevated *testosterone* concentrations in plasma—such as observed in males compared to females—is a susceptibility factor for heat stress^60^. In humans, testosterone increases during acute exertion in both females and males^61^. In our study, testosterone was a sex-independent a good discriminator between environmental and active heat stress. *Testosterone* intensities allowed differentiation between the acclimatization phase and stress PC. *Testosterone* in sweat is therefore a potential biomarker candidate for the identification and monitoring of active heat strain—for example during exertion, when heat stroke can occur even at low AT^7^.

*Cortisol* is one of the most prominent stress markers, in particular during exposure to heat^25^. The detectability of *cortisol* in sweat collected during reduced-heat-stress conditions was low; however, even at the very reduced sweat rates during the acclimatization phase, *cortisol* was occasionally detectable at higher concentrations (which might have resulted from a stressful arrival at the study site). An inverse relation between *cortisol* concentrations in sweat and indicators of moderate heat strain was observed. However, it did not withstand multivariate analysis. *Cortisol*’s role in the stress reaction—including during heat stress—is undisputed, but within this study setting, with the application of mostly only moderate heat stress, *cortisol* concentrations in sweat did not discriminate between heat stressors sensitively. *Cortisol*’s overall detectability was associated with (i) the physiological strain intensity determined by estimated whole-body sweat loss and (ii) exertion. It remains uncertain whether the quantitative assessment of *cortisol* in sweat provides additional information useful in heat-strain monitoring. Still, the qualitative binary assessment correlates with heat-stress intensity as assessed through whole-body sweat loss estimation.

*Fibromodulin* is involved in the cross-connection of *collagen*^62^, which is associated with enhanced stress resistance in *Caenorhabditis elegans*^63,64^. *Glutathione synthetase* is a key enzyme in the synthesis of glutathione, an important molecule in the protection against oxidants, and involved in plants’ heat adaptation. Low glutathione mutants fail to adapt to heat stress^65^, and the exogen supply of glutathione provides demonstrable protection against heat stress and water deficit damages in wheat^66^. In our study, differentiation between the various heat stressors was not fully possible through analysis of *glutathione synthetase*. This finding may have been influenced by a lot of missing data in this moderate heat-stress setting. However, based on its function in plants and humans, *glutathione synthetase* remains an interesting candidate—especially as *glutathione* is delivered into the circulatory system through muscle activity^67^ and physical fitness does improve heat susceptibility.

In plants, *catalase2* plays a crucial role in heat tolerance^68^. Catalase activity has been shown to be upregulated after exposure to moderate heat stress in wheat^69^ and mites^70^. Catalase activity has been shown to be affected by heat in aged rats but not in young ones, suggesting an aging affinity^71^. *Glutamylamine cyclotransferase* is one of the most heat-inhibited genes in embryos of the zebra fish^72^. In terms of *serpin B6*, it has been shown that intracellular serpins demonstrated protection from heat shock and oxidative stress induced by lysosomal injury in *C. elegans*^73^. In this study, all three proteomic markers—*catalase2*, *glutamylamine cyclotransferase*, and *serpin B6*—allowed significant differentiation between high and low environmental heat-stress levels.

The study has limitations which need consideration. The study was conducted under conditions of moderate heat stress. None of the participants reached the abort criteria (CBT = 39 °C^74^ and/or predefined maximal personal HR derived from Miller et al.^75^.), nor did any withdraw due to symptoms of heat stress. In the absence of the immediate clinical relevance of the applied heat stress, it remains to be demonstrated that the identified biomarker candidates will ultimately provide added value. While the participants were exposed to different isolated heat stressors, it is challenging to translate this setting into application in complex real life. However, it still provides hints as to each heat stressor’s impact and whether their quantitative comparison is feasible. This may enable the development of more targeted and person-centered management strategies for exposure to heat stress.

Especially during the RH visit, but also during the other visits’ acclimatization and cool-down phases, sweat rates were low. This led to a high percentage of missing data for the proteomic analysis, reducing the interpretability of the data. In future investigations, this might be addressed by either exposing participants to higher levels of heat stress and/or actively inducing local sweat glands through pilocarpine iontophoresis to generate larger sweat sample volumes^76^. However, a change in the sampling method would affect the composition of the collected sweat, and this would need to be considered^28^.

Like most biomarker candidates in sweat^77^, biomarkers’ partitioning mechanisms into sweat are not yet fully understood. During exercise, sweating facilitates the loss of AAs^78^. Parts of AA concentrations in sweat arise through the penetration of free AAs from plasma into the sweat glands, where they pair with AA residual concentrations from earlier sweating episodes^79^. Additionally, AAs in sweat are secreted from epithelial cells and serve as local moisturizers^80,81^. Further, it has been hypothesized that free AAs are released into sweat in combination with Mg^2+^, K^+^, and Ca^2+^ to facilitate resorption of Na^+^ and Cl^-^^82^. While some AAs have been shown to discriminate between heat stressors, it remains unclear if these AAs serve as surrogate markers for the reuptake of electrolytes^82^ or the sweat rate. However, there is a high potential that AA in sweat may reflect metabolic processes involved in thermoregulation.

Further, only 20 healthy participants aged 18–40 years were included in the study, and this hardly represents the diversity of society at large. It is also important to note that heat stress in real life is more complex than in a standardized lab setting, often involving multiple, concurrent heat stressors.

A couple of things need to be considered for clinical translation of the results. A more in-depth understanding of the correlations and causalities between the biomarker candidates and the heat stressors within a more inclusive and diverse population and research setting is needed. For this, clinical lab and field studies are needed to confirm a biomarker candidate’s clinical value in humans of different ages^64^ and with different comorbidities, among other factors. A thoroughly controlled clinical trial needs to be set up that complies with the highest ethical standards but allows the investigation of candidates as they approach clinically relevant abortion criteria—such as a CBT of 39 °C or the beginning of heat-strain symptoms such as dizziness. Biomarker candidates’ intensities can then be set into relation with various endpoints such as the time until visit abortion, symptoms of heat illness, and additional heat-strain markers.

A key element required for successful long-term clinical translation is the development of appropriate wearable sensing platforms to continuously, lab-independently, and non-invasively analyze sweat directly on the skin^26^. These must enable the generation of time-series data in high resolution to predict health outcomes, detect, and diagnose heat stress, but also to monitor treatment effectiveness, and recovery. The field of wearable biosensors for sweat analysis has recently seen significant developments. Research-grade wearable devices are now available to determine sweat rate and electrolyte concentrations in sweat^22^ and to detect metabolites such as AAs^30^, cortisol^32^, steroid hormones^31^, and proteins^83^. Combining sensing modules allows for the detection of various stress sources based on physical and biochemical measurements combined with artificial intelligence^84^. Crucial here will be the thorough validation of the sensors, either as in-vitro devices (sweat-based sensors) and as medical devices (for physical measurements, with the software viewed as a medical device as well). In the context of this study and once fully devised, these sensing platforms have significant potential to personalize heat stress management in occupational health. They could optimize work performance and general health, support evidence-based heat acclimatization and reduce the risk of injury. Further, these heat strain sensing platform potentially provide information about the biological age of a person^64^.

## Conclusion

This personalized study has identified novel molecular biomarker candidates in human sweat that can be employed to monitor and differentiate between heat stressors, thereby extending heat-strain monitoring beyond biophysics. Most of the identified biomarker candidates have already been investigated in plants and animals exposed to heat stress and thus harbor great potential for significantly advancing heat-strain monitoring. The identified biomarker candidates could address key challenges in the management of heat exposure. For instance, the biomarker candidates may provide more differentiated information about sex differences (based on *leucine*) and the impact of comorbidities (derived from *tryptophan* and its *metabolites*), CBT (based on *citrulline*), age (derived from *catalase2*), and heat adaption (derived from *glutathione synthetase*). These heat-strain biomarkers are especially promising due to the non-invasiveness of sweat collection and the possibility of automatic, continuous, and lab-independent monitoring through on-skin sweat-analyzing wearable devices.

## Materials and methods

### Study design and setting

The study was designed as a single-center, personalized, cross-sectional, cross-over, observational feasibility study with randomized study visits. The randomization of the study visit sequence was electronically generated by a person not involved in the recruitment and enrolment process using Excel (Microsoft Corporation, Redmond, USA). This sequence was loaded into an online study database (REDCap® – Research Electronic Data Capture; v12.4.17) for automatic unblinded allocation. Participants and the public were not involved in the study design.

The study was conducted at the Heat and Humidity Lab in Grenchen, Switzerland, from November 2022 to February 2023. Healthy study participants between the ages of 18 and 40 years were recruited from Swiss universities. The inclusion and exclusion criteria can be found in Supplemental Material 3. Additional requirements were that participants not consume alcohol or engage in excessive activity in the 24 h prior to their visits. To ensure adequate hydration status, patients were asked to drink 500 ml of plain water in the evenings before visits 2 through 4 and an additional 500 ml two hours before visits 2 through 4. In the two hours before the visits, participants were not to consume coffee or apply any skin creams. In the last hour before their visits, participants were not to eat. Before the start of visits 2 through 4, urine gravity was measured. The study was initiated only if the urine gravity was less than 1.020.

During the visits, participants wore standardized cotton clothing (0.61 clo). During the first visit, a structured assessment of the physical fitness of each participant was made. For this, $\dot{{VO}}$_2max_ was determined using the Douglas Bag method^85^. Between the first and second visit, the RH_CB_ were measured during everyday life at home at the same time of day as the planned visits using a sensor (iButton® Hygrochron; Thermodata Viewer v3.2.12) worn around the neck. The mean T_CB_ and RH_CB_ served as the personalized baseline conditions of the Heat and Humidity Lab during each accolimatization phase. Visits 2 through 4 were conducted at the same time of day. Each of visits 2 through 4 consisted of four phases, namely (i) the acclimatization phase (baseline AT and RH); (ii) the heat-stress phase (either increased AT ( + 10 °C), increased RH ( + 40%), or E (1 W/kg bodyweight)); (iii) the heat stress plus additional protective clothing phase (2 clo; +PC); and (iv) the cool-down phase (baseline AT and RH). Each phase lasted 30 min. During visit 5, it was confirmed that all e-Celsius pills ingested during visits 2 through 4 had been fully excreted.

### Ethical considerations

This observational study was approved by the Ethical Committee of Northwestern Switzerland (EKNZ ID 2022-01325). All participants provided informed consent before study enrolment. All ethical regulations relevant to human research participants were followed. The study was retrospectively registered at ClinicalTrial.gov (NCT05622188).

### Study Objectives

The aim of this study was to explore heat strain’s metabolomic and proteomic molecular signatures in sweat to identify novel biomarker candidates for health management in conditions of elevated heat. The study was embedded into a physiological validation study of a novel wearable device to monitor heat strain.

### Sampling and measurements at the heat and humidity lab

HR was continuously measured by a 3-lead holter ecg (Bittium® Faros 360; Bittium cardiac navigator software v1.5.6). Ingestion of an e-Celsius pill (eCelsius Medical capsule (BodyCAP®; monitor firmware v6.1.0 and ePerformance Manager v1.4.2)) allowed for continuous monitoring of CBT in the intestine. The participants were instructed to swallow the e-Celsius pill 6 h before the start of each visit to ensure that it had passed the stomach to reduce the impact of water intake during the study visit. Local sweat rates were measured using technical absorbents (TA; 3 M® Tegaderm+Pad – 6 ×10 cm (Sweat sampling pad 2.5 × 6 cm)) weighed by an electronic scale with a precision of 0.001 g (Mettler and Toledo®, Switzerland) before and after application. Before the application of each TA the relevant skin area was cleaned using an isopropyl-based disinfectant (SELEFA®). TAs were applied 3 cm below the mamilla on both sides of the anterior thorax, with two TAs per phase (one TA for metabolomic and one for proteomic analysis). The sample site was chosen because the wearable device, which has been physiologically validated, is attached with a chest strap. Local sweat rate was assessed once per phase for a total of four measurements per visit. After the sweat-rate assessment, the TAs’ pads were removed from the plasters and stored in Eppendorf® tubes in the freezer at −20 °C until molecular analysis. Whole-body sweat loss was estimated for each of visits 2 through 4. For this, the naked body weight of each participant was assessed before and at the end of the visit using a body scale with a precision of 100 g (Seca®, Switzerland). Drinking plain water was allowed. However, drinking and excretion of urine or stool were protocolled and included in the assessment. Participants’ hydration status was assessed through a handheld urine gravity device (Urine Specific Gravity PEN-Urine S. G., Atago®). The ambience in the heat and humidity lab was guided and monitored through a heat-stress wet-bulb globe temperature meter (TM-188D; Tenmars®).

### Metabolomic analysis

#### Liquid Chromatography Mass spectrometry (LC-MS/MS) sample preparation

The TA was transferred into a cotton swab–free Salivette® tube (Sarstedt) and 2 ml of pre-cooled extraction buffer (80% methanol containing 0.5 fmol/µl cortisol D4 and 0.5 fmol/µl cortisone 13 C) was added. After 30 min incubation at −20 °C and 4 min centrifugation at 4 °C and 1000 g, 1.8 ml of metabolite extract for each sample was retrieved in 2-ml test tubes. The extracts were dried under nitrogen flow by means of a sample concentrator (Techne®) before being stored at −80 °C until data acquisition. Prior to LC-MS analysis, extracts were reconstituted in 100 µl of 50% methanol, centrifuged at 10000 g for 10 min and transferred to sample vials (Total Recovery Glass Vial, Waters). Study-specific quality control pools were prepared by pooling 10 µl of each extract belonging to the same stress phase or stressor.

#### LC-MS/MS data acquisition

LC-MS analysis of metabolomics samples was performed on a Q-Exactive (Thermo Fisher Scientific) mass spectrometer coupled to a Thermo Vanquish LC (Thermo Scientific). Samples were separated using a 5 min gradient with a constant flow of 0.8 ml/min on a C18 analytical column (ACQUITY Premier BEH C18 Column, 1.7 µm, 2.1 ×50 mm) kept at 45 °C (LC gradient: initial conditions = 10% B, 20% B at 0.13 min, 22.5% B at 0.25 min, 45% B at 2.63 min, 95% B at 3 min, 95% B at 3.88 min, 10% B at 4.03 min, 10% B at 5 min; A buffer: H2O, 0.1% formic acid (FA), B buffer: Acetonitrile, 0.1% FA).

In the untargeted metabolomics approach, every sample was measured twice with and without fragmentation. In the first measurement, a combination of full-scan and selected-ion monitoring (SIM) was performed as follows: MS spectra were acquired in Full MS mode in the time intervals 0–0.8 min and 1.5–5 min (positive mode, scan range: m/z 70–1050, 70000 resolution, AGC target 1e6, max IT 200 ms), while between 0.8 and 1.5 min MS spectra were acquired in SIM mode to target cortisol (positive mode, scan range: m/z 150–2000, 70000 resolution, AGC target 1e6, max IT 200 ms). Cortisol and cortisol-D4 were specified in an inclusion list, m/z 363.212660 and 367.24170, respectively.

In the second measurement, a combination of data-dependent acquisition (DDA) and parallel reaction monitoring (PRM) was performed in the same run as follows: MS spectra were acquired in Full MS mode in the time intervals 0–0.8 min and 1.5–5 min (positive mode, scan range: m/z 70–1050, 70000 resolution, AGC target 1e6, max IT 200 ms), followed by ddMS2 (positive mode, scan range: m/z 200–2000, 17500 resolution, AGC target 1e5, max IT 50 ms, normalized collision energy 10, 25 and 35). In the time interval 0.8–1.5 min, MS spectra were acquired in targeted PRM mode (positive mode, scan range: m/z 150–2000, 17500 resolution, AGC target 2e5, max IT 100 ms, NCE:35). The mass spectrometry data were handled and stored using the local laboratory information management system B-fabric^86^ and all relevant data were deposited to the Mass Spectrometry Interactive Virtual Environment.

#### Data processing and metabolite identification

The MS data generated with the untargeted approach were processed by means of the commercial software Compound Discoverer 3.3 (Thermo Fisher Scientific), following the untargeted metabolomics data-processing workflow described in the Supplemental Materials 4. The modular workflow includes spectra selection, retention times alignment, compound detection and grouping, gap filling, background filtering, and normalization (data are constant median normalized, assuming that the median intensity of all detected metabolites should be the same in all samples). mzCloud and mzVault were used to score fragmentation patterns and assign MS2-based identities to the features. A filtering process was performed, leading to the manually annotated compound table, where each feature is annotated with the highest level of confidence. Filtering parameters used were the following:

Signa/noise > 3, mzCloud or mzVault match >50, ppm mass error within +/- 5ppm., match with *in house* developed MS1_RT library within +/- 10 sec., chromatographic peak and MS2 spectra quality. Consideration was also given to the biological context, leading to a classification of the identified molecules reflected in the tags associated with each compound in the manually annotated table. Cortisol quantification was performed following a targeted approach by means of the commercial software QuanBrowser (Thermo Fisher Scientific). Quantification was achieved by combining the peak intensities of a spiked-in labelled internal standard (cortisol-D4, Supelco) and a 9 levels external calibration curve of cortisol SRM (Cerilliant) from the SIM and the PRM data. In PRM quantification, two fragments were monitored by Quanbrowser for cortisol (363.2166) and D4 cortisol (367.2147).

Detectability (lower limit of detection) of cortisol was determined using a calibration curve of cortisol standard measured in the same batch as the samples. The calibration curve ranged from 5-500 fmoles injected into the column. The lower limit of detection (LOD) and lower limit of quantification were determined using this curve with the criteria for LOD as a detectable peak for cortisol (also corresponds sample to blank ratio is *>* 3 as no detectable cortisol peak was present in the blank).

### Proteomic analysis

#### LC-MS/MS sample preparation

Half of each TA was transferred into a filter plate (Porvair Sciences, 96-well, UHMW PE 25 µm, 800 µl/well) and 400 µl of 50 mM triethylamoniumbicarbonat (TEAB) buffer (pH 8.2) were added. Samples were reduced with 2 mM TCEP (tris(2-carboxyethyl) phosphine) and alkylated with 15 mM chloroacetamide at 30 °C for 45 min.

500 ng of Sequencing Grade Trypsin (Promega) were dissolved in 100 µl of 50 mM TEAB buffer and added for digestion carried out overnight at 37 °C.

Filter plates were centrifuged at 3000 g for 10 min to collect digested peptides. The solution was dried to completeness and re-solubilized in 80 µl of MS sample buffer (3% acetonitrile, 0.1% formic acid). 20 µl of each sample were loaded on an Evotip (Evosep Biosystems) according to the manufacturer’s protocol.

#### LC-MS/MS data acquisition

MS analysis of proteomics samples was performed on a timsTOF Pro (Bruker) coupled to an Evosep One (EvoSep Biosystems). Samples were separated using the 30 SPD method while keeping the analytical column (PepSep C18, 15 cm×150 µm, 1.5 µm) at 40 °C. MS scans were acquired from m/z 100 to m/z 1700 with an inverse mobility ramp [1/K0] from 0.60 Vs/cm2 to 1.60 Vs/cm2 (ion accumulation and ramp time both set at 100 ms). MS2 scans were acquired in diaPASEF mode (data independent acquisition Parallel Accumulation Serial Fragmentation); one MS scan was followed by 16 PASEF cycles between m/z 400 and m/z 1200 with overlapping isolation windows of m/z 26, covering 2 ×0.30 [1/K0] ion mobility windows in the range of 0.60 Vs/cm2 to 1.42 Vs/cm2. Singly charged ions were excluded using the ion mobility polygon filter mask.

The mass spectrometry data were handled using the local laboratory information management system B-fabric as described for the metabolomic analysis. All relevant data have been deposited on the ProteomeXchange Consortium via the PRIDE.

#### Protein identification and quantification

The acquired MS data were processed for identification and quantification using the DIA-NN 1.8.1 software suite [Demichev, V., Messner, C.B., Vernardis, S.I. et al. DIA-NN: neural networks and interference correction enable deep proteome coverage in high throughput. *Nat Methods* **17**, 41–44 (2020). 10.1038/s41592-019-0638-x]. Spectra were searched against the human uniprot proteome database (UP000005640) containing common protein contaminants. Carbamidomethylation of cysteines and methionine oxidation were included as fixed and variable modifications, respectively. Enzyme specificity was set to trypsin/P, allowing a minimum peptide length of seven amino acids and a maximum of two missed cleavages. The search was conducted in library-free mode with default settings. Intensities of protein groups were calculated as sums of the intensities of the three most abundant precursors identified with a q-value lower than 0.01 and with normalization disabled.

### Statistical analysis

#### Screening of metabolites/proteins with differential stress trajectories

The question here is whether metabolites display differences in response to stress for various comparisons of interest^1^: ambient-temperature and relative-humidity stress vs. exertion (and in combination with protective clothing)^2^; high WBGT (ambient temperature stress) vs. low WBGT (relative humidity stress); and^3^ ambient heat stress vs. active heat stress. In order to screen promising metabolites, linear models were fitted with the relative intensity trajectory of each metabolite as the outcome and the stress-phase/+PC variable as explanatory predictor. Further adjustment was not performed because the data consisted of relative quantifications already considered to be comparable within and across subjects. Contrast matrices were constructed to address the specific hypotheses of interest and to obtain estimates. Multiplicity-corrected values obtained using the Benjamini-Hochberg method (per question) for metabolites and proteins separately are also reported. For each comparison, a table of the top ten results gives the metabolite ID and name, effect estimate, standard error, test statistic, and *p*-value from the regression. The top metabolites were obtained by filtering by lowest *p*-value through metabolites with significant trajectory differences between both acclimation and stress and acclimation and stress + clothing. The analysis and reporting are similar for proteins. However, some data was missing among the protein concentrations. Only proteins with less than 50% missing data were analyzed. Missing values were imputed with half the lowest value for a given protein.

#### Association between biomarkers and vital parameters

To explore possible associations between the metabolomic and proteomic markers and the vital parameters CBT and HR, linear models were fitted with adjustments for stress type and phase. To deal with missing proteomic data, only proteins with less than 50% missing values were analyzed. Additionally, half the lowest observed value was imputed per protein. Results were filtered down to models with significant Wald test statistics for the CBT or HR coefficient respectively. The metabolites and proteins with statistically significant associations with vital parameters are presented in tables with the effect estimate, standard error, test statistic, and *p*-value from the regression.

#### Cortisol detection

Cortisol was expected to be detected more often under some types of stress than others. To summarize the detection of cortisol, the proportion of subjects in which cortisol could be detected was estimated (with 95% Wilson confidence intervals) per stress (visit type) and phase (acclimatization, stress, stress+ protective clothing, cool-down). This summary is presented as a table. Additionally, a logistic regression model was fitted with the binary detection indicator as the outcome and stress type as variable of interest. Tukey’s single-step multiple comparison procedure was applied to the regression model to obtain odds ratios for all stress-pair comparisons. These are shown in a table with the coefficients and standard errors on the log scale, and corresponding *p*-values.

#### Relationship between cortisol values and vital parameters

A potential relationship between cortisol values and (i) vital parameters and (ii) heat-shock protein values was of interest. First, scatter plots of log cortisol values versus each of the other parameters (CBT, HR, log plaster-based local sweat rate, log heat-shock protein concentration) were created. CBT and HR were measured every 30 s (as opposed to once per phase), so these measures were summarized by the means of the values in the final 5 min of the stress phase. Additionally, linear regression models were fitted with log cortisol values as the outcome. First, univariable models were fitted with each of the other parameters separately, and then a multivariable model was fitted with all the other parameters. The results are presented as a table of coefficients with 95% confidence intervals and Wald *p*-values.

### Reporting summary

Further information on research design is available in the Nature Portfolio Reporting Summary linked to this article.

## Supplementary information

Supplemental Material

Description of Additional Supplementary Files

Supplementary Data 1

Reporting Summary

## Data Availability

Source data is available from the, corresponding author on reasonable request. Source data behind the graphs can be found in - Supplementary data 1 file. Proteomic mass spectrometry data is published and accessible from Proteomics Identification Database (https://www.ebi.ac.uk/pride/) with the ID: PXD053287. Metabolomic data is published and accessible from Mass Spectrometry Interactive Virtual Environment (https://massive.ucsd.edu/ProteoSAFe/) with the ID: MassIVE MSV000096263.
