# Supplementary material for: The molecular signature of heat stress in sweat reveals non-invasive biomarker candidates for health monitoring
Source: Commun Biol. 2025 Apr 23;8:650. doi: 10.1038/s42003-025-08080-1 (PMC12019370; doi:10.1038/s42003-025-08080-1)
Supplement: Supplementary file 1 — Supplemental Material [file 42003_2025_8080_MOESM1_ESM.pdf]

Supplemental Material 1. Local and whole-body estimate sweat rate assessment.

heatstress_type	phase	mean local sweat loss (g)	sd local sweat loss (g)	mean local sweat rate (g*m^-2 *h^-1)	sd local sweat rate (g*m^-2 *h^-1)	mean whole body sweat loss per body surface (L/m^2)	sd whole body sweat loss per body surface (L/m^2)
Ambient Temperature	Acclim	0.028	0.026	37.324	34.658		
Ambient Temperature	Stress	0.139	0.074	185.287	98.642		
Ambient Temperature	Stress+Protective Clothing	0.272	0.128	362.576	170.624		
Ambient Temperature	Cool	0.052	0.031	69.316	41.323	0.16	0.05
Relative Humidity	Acclim	0.018	0.017	23.994	22.661		
Relative Humidity	Stress	0.033	0.02	43.989	26.66		
Relative Humidity	Stress+Protective Clothing	0.061	0.048	81.313	63.984		
Relative Humidity	Cool	0.029	0.02	38.657	26.66	0.07	0.03
Exertion	Acclim	0.025	0.024	33.325	31.992		
Exertion	Stress	0.422	0.169	562.526	225.277		
Exertion	Stress+Protective Clothing	0.967	0.64	1289.011	853.12		
Exertion	Cool	0.076	0.033	101.308	43.989	0.3	0.07

Supplemental Material 2. Wet-bulb globe temperature during the visits.

Stress	Phase	Estimate WBGT (°C)	Difference	CI	p
Ambient Temperature	Acclimation	23.24			
Ambient Temperature	Stress	31.26	8.02	(6.95 to 9.09)	< 0.0001

Ambient Temperature	Stress+Protective Clothing	31.39	8.15	(7.08 to 9.22)	< 0.0001
Ambient Temperature	Cool	23.28	0.04	(-1.03 to 1.11)	0.95
Relative Humidity	Acclimation	23.16			
Relative Humidity	Stress	27.74	4.58	(3.51 to 5.65)	< 0.0001
Relative Humidity	Stress+Protective Clothing	27.91	4.75	(3.68 to 5.82)	< 0.0001
Relative Humidity	Cool	23.31	0.15	(-0.92 to 1.22)	0.78
Exertion	Acclimation	23.29			
Exertion	Stress	23.53	0.23	(-0.86 to 1.33)	0.68
Exertion	Stress+Protective Clothing	23.42	0.13	(-0.97 to 1.23)	0.82
Exertion	Cool	23.04	-0.25	(-1.35 to 0.85)	0.66

Supplemental Material 3. Inclusion and exclusion criteria.

**Inclusion criteria**
- Healthy participant, able to give consent<br>- Non-athlete (<4h sport/week)<br>- BMI<30 (non-obese)<br>- aged 18-40<br>- German speaking, or fluent in German
**Exclusion criteria**
- Pregnancy, breastfeeding<br>- Regular medication intake (excluding birth control pill)<br>- Intake of drugs and/or daily alcohol consumption<br>- Fever or symptoms of an acute infection (cough, shortness of breath, sore throat, loss of taste)<br>- Active smoking or history of smoking <9months ago<br>- Mobility impairment

- Travelled (<1month ago) to a warm/hot temperature zone and stayed for >6 days
- Any chronic conditions such as: high blood pressure, diabetes mellitus, immuno-deficiencies, sweat disorders such as anhidrosis
- Color blindness
- Weight <40kg
- Diverticulum or obstructions of the gastrointestinal tract (including motility disorders, swallowing disorders) as well as major abdominal surgery (e-Celsius pill)
- Need of exposure to strong electromagnetic fields during trial participation, above all MRI examinations (e-Celsius pill)

Supplemental Material 4. Description of the untargeted metabolomics data-processing workflow.

CD workflow o31243

Search name: p29943_o31243_Sweat_Metabolomics_Untargeted_MS2

Search description: Untargeted Metabolomics workflow: Find and identify the differences between samples.

- Performs retention time alignment, unknown compound detection, and compound grouping across all samples. Predicts elemental compositions for all compounds, fills gaps across all samples, and hides chemical background (using Blank samples). Identifies compounds using mzCloud (ddMS2) and ChemSpider (formula or exact mass). Also performs similarity search for all compounds with ddMS2 data using mzCloud. Applies mzLogic algorithm to rank order ChemSpider results. Maps compounds to biological pathways using Metabolika. Applies QC-based batch normalization if QC samples are available. Calculates differential analysis (t-test or ANOVA), determines p-values, adjusted p-values, ratios, fold change, CV, etc.).

Search date: 03/08/2023 14:04:02

Created with Discoverer version: 3.3.1.111

[Input Files (6)]

-->Select Spectra (33)

[Select Spectra (33)]

-->Align Retention Times (ChromAlign) (46)

[Align Retention Times (ChromAlign) (46)]

-->Detect Compounds (49)

[Detect Compounds (49)]

-->Group Compounds (31)

[Group Compounds (31)]

-->Fill Gaps (32)

-->Assign Compound Annotations (25)

-->Search mzCloud (42)

-->Search mzVault (51)

-->Predict Compositions (40)

-->Search ChemSpider (41)

-->Search Mass Lists (50)

[Fill Gaps (32)]

-->Mark Background Compounds (28)

[Mark Background Compounds (28)]

-->Normalize Areas (52)

[Search ChemSpider (41)]

-->Apply mzLogic (48)

[Search Mass Lists (50)]

-->Apply mzLogic (48)

[Normalize Areas (52)]

[Assign Compound Annotations (25)]

[Search mzCloud (42)]

[Search mzVault (51)]

[Predict Compositions (40)]

[Apply mzLogic (48)]

[Descriptive Statistics (54)]

[Differential Analysis (55)]

Processing node 6: Input Files

Input Data:

- File Name(s) (Hidden):

D:\o31243\tSIM_data\20230614_SIM_Blank2.raw

D:\o31243\tSIM_data\20230614_SIM_Blank3.raw

D:\o31243\tSIM_data\20230614_SIM_Blank4.raw

D:\o31243\tSIM_data\20230614_SIM_Blank5.raw

D:\o31243\tSIM_data\20230614_SIM_Blank6.raw

D:\o31243\tSIM_data\20230614_SIM_Blank7.raw

D:\o31243\tSIM_data\20230614_SIM_Blank8.raw

D:\o31243\tSIM_data\20230614_SIM_Blank9.raw

D:\o31243\tSIM_data\20230614_SIM_Blank10.raw

D:\o31243\tSIM_data\20230614_SIM_QCpool_1.raw

D:\o31243\tSIM_data\20230614_SIM_QCpool_2.raw

D:\o31243\tSIM_data\20230614_SIM_QCpool_3.raw

D:\o31243\tSIM_data\20230614_SIM_QCpool_4.raw

D:\o31243\tSIM_data\20230614_SIM_QCpool_Steroids_mix_1fmol_1.raw

D:\o31243\tSIM_data\20230614_SIM_QCpool_Steroids_mix_1fmol_3.raw

D:\o31243\tSIM_data\20230614_SIM_QCpool_Steroids_mix_5fmol_1.raw

D:\o31243\tSIM_data\20230614_SIM_QCpool_Steroids_mix_5fmol_3.raw

D:\o31243\tSIM_data\20230614_SIM_QCpool_Steroids_mix_10fmol_1.raw

D:\o31243\tSIM_data\20230614_SIM_QCpool_Steroids_mix_10fmol_3.raw

D:\o31243\tSIM_data\20230614_SIM_QCpool_Steroids_mix_25fmol_1.raw

D:\o31243\tSIM_data\20230614_SIM_QCpool_Steroids_mix_25fmol_3.raw

D:\o31243\tSIM_data\20230614_SIM_QCpool_Steroids_mix_50fmol_1.raw

D:\o31243\tSIM_data\20230614_SIM_QCpool_Steroids_mix_50fmol_3.raw

D:\o31243\tSIM_data\20230614_SIM_QCpool_Steroids_mix_100fmol_1.raw

D:\o31243\tSIM_data\20230614_SIM_QCpool_Steroids_mix_100fmol_3.raw

D:\o31243\tSIM_data\20230614_SIM_QCpool_Steroids_mix_250fmol_1.raw

D:\o31243\tSIM_data\20230614_SIM_QCpool_Steroids_mix_250fmol_3.raw

D:\o31243\tSIM_data\20230614_SIM_Steroids_mix_1_25fmol_1.raw

D:\o31243\tSIM_data\20230614_SIM_Steroids_mix_1_25fmol_2.raw

D:\o31243\tSIM_data\20230614_SIM_Steroids_mix_1_25fmol_3.raw

D:\o31243\tSIM_data\20230614_SIM_Steroids_mix_2_5fmol_1.raw

D:\o31243\tSIM_data\20230614_SIM_Steroids_mix_2_5fmol_2.raw

D:\o31243\tSIM_data\20230614_SIM_Steroids_mix_2_5fmol_3.raw

D:\o31243\tSIM_data\20230614_SIM_Steroids_mix_5fmol_1.raw

D:\o31243\tSIM_data\20230614_SIM_Steroids_mix_5fmol_2.raw

D:\o31243\tSIM_data\20230614_SIM_Steroids_mix_5fmol_3.raw

D:\o31243\tSIM_data\20230614_SIM_Steroids_mix_10fmol_1.raw

D:\o31243\tSIM_data\20230614_SIM_Steroids_mix_10fmol_2.raw

D:\o31243\tSIM_data\20230614_SIM_Steroids_mix_10fmol_3.raw

D:\o31243\tSIM_data\20230614_SIM_Steroids_mix_20fmol_1.raw

D:\o31243\tSIM_data\20230614_SIM_Steroids_mix_20fmol_2.raw

D:\o31243\tSIM_data\20230614_SIM_Steroids_mix_20fmol_3.raw

D:\o31243\tSIM_data\20230614_SIM_Steroids_mix_50fmol_1.raw

D:\o31243\tSIM_data\20230614_SIM_Steroids_mix_50fmol_2.raw

D:\o31243\tSIM_data\20230614_SIM_Steroids_mix_50fmol_3.raw

D:\o31243\tSIM_data\20230614_SIM_Steroids_mix_100fmol_1.raw

D:\o31243\tSIM_data\20230614_SIM_Steroids_mix_100fmol_2.raw

D:\o31243\tSIM_data\20230614_SIM_Steroids_mix_100fmol_3.raw

D:\o31243\tSIM_data\20230614_SIM_Steroids_mix_250fmol_1.raw

D:\o31243\tSIM_data\20230614_SIM_Steroids_mix_250fmol_2.raw

D:\o31243\tSIM_data\20230614_SIM_Steroids_mix_250fmol_3.raw

D:\o31243\tSIM_data\20230614_SIM_Steroids_mix_500fmol_1.raw

D:\o31243\tSIM_data\20230614_SIM_Steroids_mix_500fmol_2.raw

D:\o31243\tSIM_data\20230614_SIM_Steroids_mix_500fmol_3.raw

D:\o31243\tSIM_data\20230614_SIM_TubeID_1_Sample_01-02-12.raw

D:\o31243\tSIM_data\20230614_SIM_TubeID_2_Sample_01-02-22.raw

D:\o31243\tSIM_data\20230614_SIM_TubeID_3_Sample_01-02-32.raw

D:\o31243\tSIM_data\20230614_SIM_TubeID_4_Sample_01-02-42.raw

D:\o31243\tSIM_data\20230614_SIM_TubeID_5_Sample_01-03-12.raw

D:\o31243\tSIM_data\20230614_SIM_TubeID_6_Sample_01-03-22.raw

D:\o31243\tSIM_data\20230614_SIM_TubeID_7_Sample_01-03-32.raw

D:\o31243\tSIM_data\20230614_SIM_TubeID_8_Sample_01-03-42.raw

D:\o31243\tSIM_data\20230614_SIM_TubeID_9_Sample_01-04-12.raw

D:\o31243\tSIM_data\20230614_SIM_TubeID_10_Sample_01-04-22.raw

D:\o31243\tSIM_data\20230614_SIM_TubeID_11_Sample_01-04-32.raw

D:\o31243\tSIM_data\20230614_SIM_TubeID_12_Sample_01-04-42.raw

D:\o31243\tSIM_data\20230614_SIM_TubeID_13_Sample_02-02-12.raw

D:\o31243\tSIM_data\20230614_SIM_TubeID_14_Sample_02-02-22.raw

D:\o31243\tSIM_data\20230614_SIM_TubeID_15_Sample_02-02-32.raw

D:\o31243\tSIM_data\20230614_SIM_TubeID_16_Sample_02-02-42.raw

D:\o31243\tSIM_data\20230614_SIM_TubeID_17_Sample_02-03-12.raw

D:\o31243\tSIM_data\20230614_SIM_TubeID_18_Sample_02-03-22.raw

D:\o31243\tSIM_data\20230614_SIM_TubeID_19_Sample_02-03-32.raw

D:\o31243\tSIM_data\20230614_SIM_TubeID_20_Sample_02-03-42.raw

D:\o31243\tSIM_data\20230614_SIM_TubeID_21_Sample_02-04-12.raw

D:\o31243\tSIM_data\20230614_SIM_TubeID_22_Sample_02-04-22.raw

D:\o31243\tSIM_data\20230614_SIM_TubeID_23_Sample_02-04-32.raw

D:\o31243\tSIM_data\20230614_SIM_TubeID_24_Sample_02-04-42.raw

D:\o31243\tSIM_data\20230614_SIM_TubeID_25_Sample_03-02-12.raw

D:\o31243\tSIM_data\20230614_SIM_TubeID_26_Sample_03-02-22.raw

D:\o31243\tSIM_data\20230614_SIM_TubeID_27_Sample_03-02-32.raw

D:\o31243\tSIM_data\20230614_SIM_TubeID_28_Sample_03-02-42.raw

D:\o31243\tSIM_data\20230614_SIM_TubeID_29_Sample_03-03-12.raw

D:\o31243\tSIM_data\20230614_SIM_TubeID_30_Sample_03-03-22.raw

D:\o31243\tSIM_data\20230614_SIM_TubeID_31_Sample_03-03-32.raw

D:\o31243\tSIM_data\20230614_SIM_TubeID_32_Sample_03-03-42.raw

D:\o31243\tSIM_data\20230614_SIM_TubeID_33_Sample_03-04-12.raw

D:\o31243\tSIM_data\20230614_SIM_TubeID_34_Sample_03-04-22.raw

D:\o31243\tSIM_data\20230614_SIM_TubeID_35_Sample_03-04-32.raw

D:\o31243\tSIM_data\20230614_SIM_TubeID_36_Sample_03-04-42.raw

D:\o31243\tSIM_data\20230614_SIM_TubeID_37_Sample_04-02-12.raw

D:\o31243\tSIM_data\20230614_SIM_TubeID_38_Sample_04-02-22.raw

D:\o31243\tSIM_data\20230614_SIM_TubeID_39_Sample_04-02-32.raw

D:\o31243\tSIM_data\20230614_SIM_TubeID_40_Sample_04-02-42.raw

D:\o31243\tSIM_data\20230614_SIM_TubeID_41_Sample_04-03-12.raw

D:\o31243\tSIM_data\20230614_SIM_TubeID_42_Sample_04-03-22.raw

D:\o31243\tSIM_data\20230614_SIM_TubeID_43_Sample_04-03-32.raw

D:\o31243\tSIM_data\20230614_SIM_TubeID_44_Sample_04-03-42.raw

D:\o31243\tSIM_data\20230614_SIM_TubeID_45_Sample_04-04-12.raw

D:\o31243\tSIM_data\20230614_SIM_TubeID_46_Sample_04-04-22.raw

D:\o31243\tSIM_data\20230614_SIM_TubeID_47_Sample_04-04-32.raw

D:\o31243\tSIM_data\20230614_SIM_TubeID_48_Sample_04-04-42.raw

D:\o31243\tSIM_data\20230614_SIM_TubeID_49_Sample_05-03-12.raw

D:\o31243\tSIM_data\20230614_SIM_TubeID_50_Sample_05-03-22.raw

D:\o31243\tSIM_data\20230614_SIM_TubeID_51_Sample_05-03-32.raw

D:\o31243\tSIM_data\20230614_SIM_TubeID_52_Sample_05-03-42.raw

D:\o31243\tSIM_data\20230614_SIM_TubeID_53_Sample_05-04-12.raw

D:\o31243\tSIM_data\20230614_SIM_TubeID_54_Sample_05-04-22.raw

D:\o31243\tSIM_data\20230614_SIM_TubeID_55_Sample_05-04-32.raw

D:\o31243\tSIM_data\20230614_SIM_TubeID_56_Sample_05-04-42.raw

D:\o31243\tSIM_data\20230614_SIM_TubeID_57_Sample_06-02-12.raw

D:\o31243\tSIM_data\20230614_SIM_TubeID_58_Sample_06-02-22.raw

D:\o31243\tSIM_data\20230614_SIM_TubeID_59_Sample_06-02-32.raw

D:\o31243\tSIM_data\20230614_SIM_TubeID_60_Sample_06-02-42.raw

D:\o31243\tSIM_data\20230614_SIM_TubeID_61_Sample_06-03-12.raw

D:\o31243\tSIM_data\20230614_SIM_TubeID_62_Sample_06-03-22.raw

D:\o31243\tSIM_data\20230614_SIM_TubeID_63_Sample_06-03-32.raw

D:\o31243\tSIM_data\20230614_SIM_TubeID_64_Sample_06-03-42.raw

D:\o31243\tSIM_data\20230614_SIM_TubeID_65_Sample_06-04-12.raw

D:\o31243\tSIM_data\20230614_SIM_TubeID_66_Sample_06-04-22.raw

D:\o31243\tSIM_data\20230614_SIM_TubeID_67_Sample_06-04-32.raw

D:\o31243\tSIM_data\20230614_SIM_TubeID_68_Sample_06-04-42.raw

D:\o31243\tSIM_data\20230614_SIM_TubeID_69_Sample_07-02-12.raw

D:\o31243\tSIM_data\20230614_SIM_TubeID_70_Sample_07-02-22.raw

D:\o31243\tSIM_data\20230614_SIM_TubeID_71_Sample_07-02-32.raw

D:\o31243\tSIM_data\20230614_SIM_TubeID_72_Sample_07-02-42.raw

D:\o31243\tSIM_data\20230614_SIM_TubeID_73_Sample_07-03-12.raw

D:\o31243\tSIM_data\20230614_SIM_TubeID_74_Sample_07-03-22.raw

D:\o31243\tSIM_data\20230614_SIM_TubeID_75_Sample_07-03-32.raw

D:\o31243\tSIM_data\20230614_SIM_TubeID_76_Sample_07-03-42.raw

D:\o31243\tSIM_data\20230614_SIM_TubeID_77_Sample_07-04-12.raw

D:\o31243\tSIM_data\20230614_SIM_TubeID_78_Sample_07-04-22.raw

D:\o31243\tSIM_data\20230614_SIM_TubeID_79_Sample_07-04-32.raw

D:\o31243\tSIM_data\20230614_SIM_TubeID_80_Sample_07-04-42.raw

D:\o31243\tSIM_data\20230614_SIM_TubeID_81_Sample_08-02-12.raw

D:\o31243\tSIM_data\20230614_SIM_TubeID_82_Sample_08-02-22.raw

D:\o31243\tSIM_data\20230614_SIM_TubeID_83_Sample_08-02-32.raw

D:\o31243\tSIM_data\20230614_SIM_TubeID_84_Sample_08-02-42.raw

D:\o31243\tSIM_data\20230614_SIM_TubeID_85_Sample_08-03-12.raw

D:\o31243\tSIM_data\20230614_SIM_TubeID_86_Sample_08-03-22.raw

D:\o31243\tSIM_data\20230614_SIM_TubeID_87_Sample_08-03-32.raw

D:\o31243\tSIM_data\20230614_SIM_TubeID_88_Sample_08-03-42.raw

D:\o31243\tSIM_data\20230614_SIM_TubeID_89_Sample_08-04-12.raw

D:\o31243\tSIM_data\20230614_SIM_TubeID_90_Sample_08-04-22.raw

D:\o31243\tSIM_data\20230614_SIM_TubeID_91_Sample_08-04-32.raw

D:\o31243\tSIM_data\20230614_SIM_TubeID_92_Sample_08-04-42.raw

D:\o31243\tSIM_data\20230614_SIM_TubeID_93_Sample_09-02-12.raw

D:\o31243\tSIM_data\20230614_SIM_TubeID_94_Sample_09-02-22.raw

D:\o31243\tSIM_data\20230614_SIM_TubeID_95_Sample_09-02-32.raw

D:\o31243\tSIM_data\20230614_SIM_TubeID_96_Sample_09-02-42.raw

D:\o31243\tSIM_data\20230614_SIM_TubeID_97_Sample_09-03-12.raw

D:\o31243\tSIM_data\20230614_SIM_TubeID_98_Sample_09-03-22.raw

D:\o31243\tSIM_data\20230614_SIM_TubeID_99_Sample_09-03-32.raw

D:\o31243\tSIM_data\20230614_SIM_TubeID_100_Sample_09-03-42.raw

D:\o31243\tSIM_data\20230614_SIM_TubeID_101_Sample_09-04-12.raw

D:\o31243\tSIM_data\20230614_SIM_TubeID_102_Sample_09-04-22.raw

D:\o31243\tSIM_data\20230614_SIM_TubeID_103_Sample_09-04-32.raw

D:\o31243\tSIM_data\20230614_SIM_TubeID_104_Sample_09-04-42.raw

D:\o31243\tSIM_data\20230614_SIM_TubeID_105_Sample_10-02-12.raw

D:\o31243\tSIM_data\20230614_SIM_TubeID_106_Sample_10-02-22.raw

D:\o31243\tSIM_data\20230614_SIM_TubeID_107_Sample_10-02-32.raw

D:\o31243\tSIM_data\20230614_SIM_TubeID_108_Sample_10-02-42.raw

D:\o31243\tSIM_data\20230614_SIM_TubeID_109_Sample_10-03-12.raw

D:\o31243\tSIM_data\20230614_SIM_TubeID_110_Sample_10-03-22.raw

D:\o31243\tSIM_data\20230614_SIM_TubeID_111_Sample_10-03-32.raw

D:\o31243\tSIM_data\20230614_SIM_TubeID_112_Sample_10-03-42.raw

D:\o31243\tSIM_data\20230614_SIM_TubeID_113_Sample_10-04-12.raw

D:\o31243\tSIM_data\20230614_SIM_TubeID_114_Sample_10-04-22.raw

D:\o31243\tSIM_data\20230614_SIM_TubeID_115_Sample_10-04-32.raw

D:\o31243\tSIM_data\20230614_SIM_TubeID_116_Sample_10-04-42.raw

D:\o31243\tSIM_data\20230614_SIM_TubeID_117_Sample_11-02-21.raw

D:\o31243\tSIM_data\20230614_SIM_TubeID_118_Sample_11-02-22.raw

D:\o31243\tSIM_data\20230614_SIM_TubeID_119_Sample_11-02-32.raw

D:\o31243\tSIM_data\20230614_SIM_TubeID_120_Sample_11-02-42.raw

D:\o31243\tSIM_data\20230614_SIM_TubeID_121_Sample_11-03-12.raw

D:\o31243\tSIM_data\20230614_SIM_TubeID_122_Sample_11-03-22.raw

D:\o31243\tSIM_data\20230614_SIM_TubeID_123_Sample_11-03-32.raw

D:\o31243\tSIM_data\20230614_SIM_TubeID_124_Sample_11-03-42.raw

D:\o31243\tSIM_data\20230614_SIM_TubeID_125_Sample_11-04-12.raw

D:\o31243\tSIM_data\20230614_SIM_TubeID_126_Sample_11-04-22.raw

D:\o31243\tSIM_data\20230614_SIM_TubeID_127_Sample_11-04-32.raw

D:\o31243\tSIM_data\20230614_SIM_TubeID_128_Sample_11-04-42.raw

D:\o31243\tSIM_data\20230614_SIM_TubeID_129_Sample_12-02-12.raw

D:\o31243\tSIM_data\20230614_SIM_TubeID_130_Sample_12-02-22.raw

D:\o31243\tSIM_data\20230614_SIM_TubeID_131_Sample_12-02-32.raw

D:\o31243\tSIM_data\20230614_SIM_TubeID_132_Sample_12-02-42.raw

D:\o31243\tSIM_data\20230614_SIM_TubeID_133_Sample_12-03-12.raw

D:\o31243\tSIM_data\20230614_SIM_TubeID_134_Sample_12-03-22.raw

D:\o31243\tSIM_data\20230614_SIM_TubeID_135_Sample_12-03-32.raw

D:\o31243\tSIM_data\20230614_SIM_TubeID_136_Sample_12-03-42.raw

D:\o31243\tSIM_data\20230614_SIM_TubeID_137_Sample_12-04-12.raw

D:\o31243\tSIM_data\20230614_SIM_TubeID_138_Sample_12-04-22.raw

D:\o31243\tSIM_data\20230614_SIM_TubeID_139_Sample_12-04-32.raw

D:\o31243\tSIM_data\20230614_SIM_TubeID_140_Sample_12-04-42.raw

D:\o31243\tSIM_data\20230614_SIM_TubeID_141_Sample_13-02-12.raw

D:\o31243\tSIM_data\20230614_SIM_TubeID_142_Sample_13-02-22.raw

D:\o31243\tSIM_data\20230614_SIM_TubeID_143_Sample_13-02-32.raw

D:\o31243\tSIM_data\20230614_SIM_TubeID_144_Sample_13-02-42.raw

D:\o31243\tSIM_data\20230614_SIM_TubeID_145_Sample_13-03-12.raw

D:\o31243\tSIM_data\20230614_SIM_TubeID_146_Sample_13-03-22.raw

D:\o31243\tSIM_data\20230614_SIM_TubeID_147_Sample_13-03-32.raw

D:\o31243\tSIM_data\20230614_SIM_TubeID_148_Sample_13-03-42.raw

D:\o31243\tSIM_data\20230614_SIM_TubeID_149_Sample_13-04-12.raw

D:\o31243\tSIM_data\20230614_SIM_TubeID_150_Sample_13-04-22.raw

D:\o31243\tSIM_data\20230614_SIM_TubeID_151_Sample_13-04-32.raw

D:\o31243\tSIM_data\20230614_SIM_TubeID_152_Sample_13-04-42.raw

D:\o31243\tSIM_data\20230614_SIM_TubeID_153_Sample_15-02-12.raw

D:\o31243\tSIM_data\20230614_SIM_TubeID_154_Sample_15-02-22.raw

D:\o31243\tSIM_data\20230614_SIM_TubeID_155_Sample_15-02-32.raw

D:\o31243\tSIM_data\20230614_SIM_TubeID_156_Sample_15-02-42.raw

D:\o31243\tSIM_data\20230614_SIM_TubeID_157_Sample_15-03-12.raw

D:\o31243\tSIM_data\20230614_SIM_TubeID_158_Sample_15-03-22.raw

D:\o31243\tSIM_data\20230614_SIM_TubeID_159_Sample_15-03-32.raw

D:\o31243\tSIM_data\20230614_SIM_TubeID_160_Sample_15-03-42.raw

D:\o31243\tSIM_data\20230614_SIM_TubeID_161_Sample_15-04-12.raw

D:\o31243\tSIM_data\20230614_SIM_TubeID_162_Sample_15-04-22.raw

D:\o31243\tSIM_data\20230614_SIM_TubeID_163_Sample_15-04-32.raw

D:\o31243\tSIM_data\20230614_SIM_TubeID_164_Sample_15-04-42.raw

D:\o31243\tSIM_data\20230614_SIM_TubeID_165_Sample_18-02-12.raw

D:\o31243\tSIM_data\20230614_SIM_TubeID_166_Sample_18-02-22.raw

D:\o31243\tSIM_data\20230614_SIM_TubeID_167_Sample_18-02-32.raw

D:\o31243\tSIM_data\20230614_SIM_TubeID_168_Sample_18-02-42.raw

D:\o31243\tSIM_data\20230614_SIM_TubeID_169_Sample_18-03-12.raw

D:\o31243\tSIM_data\20230614_SIM_TubeID_170_Sample_18-03-22.raw

D:\o31243\tSIM_data\20230614_SIM_TubeID_171_Sample_18-03-32.raw

D:\o31243\tSIM_data\20230614_SIM_TubeID_172_Sample_18-03-42.raw

D:\o31243\tSIM_data\20230614_SIM_TubeID_173_Sample_18-04-12.raw

D:\o31243\tSIM_data\20230614_SIM_TubeID_174_Sample_18-04-22.raw

D:\o31243\tSIM_data\20230614_SIM_TubeID_175_Sample_18-04-32.raw

D:\o31243\tSIM_data\20230614_SIM_TubeID_176_Sample_18-04-42.raw

D:\o31243\tSIM_data\20230614_SIM_TubeID_177_Sample_19-02-12.raw

D:\o31243\tSIM_data\20230614_SIM_TubeID_178_Sample_19-02-22.raw

D:\o31243\tSIM_data\20230614_SIM_TubeID_179_Sample_19-02-32.raw

D:\o31243\tSIM_data\20230614_SIM_TubeID_180_Sample_19-02-42.raw

D:\o31243\tSIM_data\20230614_SIM_TubeID_181_Sample_19-03-12.raw

D:\o31243\tSIM_data\20230614_SIM_TubeID_182_Sample_19-03-22.raw

D:\o31243\tSIM_data\20230614_SIM_TubeID_183_Sample_19-03-32.raw

D:\o31243\tSIM_data\20230614_SIM_TubeID_184_Sample_19-03-42.raw

D:\o31243\tSIM_data\20230614_SIM_TubeID_185_Sample_19-04-12.raw

D:\o31243\tSIM_data\20230614_SIM_TubeID_186_Sample_19-04-22.raw

D:\o31243\tSIM_data\20230614_SIM_TubeID_187_Sample_19-04-32.raw

D:\o31243\tSIM_data\20230614_SIM_TubeID_188_Sample_19-04-42.raw

D:\o31243\tSIM_data\20230614_SIM_TubeID_189_Sample_20-02-12.raw

D:\o31243\tSIM_data\20230614_SIM_TubeID_190_Sample_20-02-22.raw

D:\o31243\tSIM_data\20230614_SIM_TubeID_191_Sample_20-02-32.raw

D:\o31243\tSIM_data\20230614_SIM_TubeID_192_Sample_20-02-42.raw

D:\o31243\tSIM_data\20230614_SIM_TubeID_193_Sample_20-03-12.raw

D:\o31243\tSIM_data\20230614_SIM_TubeID_194_Sample_20-03-22.raw

D:\o31243\tSIM_data\20230614_SIM_TubeID_195_Sample_20-03-32.raw

D:\o31243\tSIM_data\20230614_SIM_TubeID_196_Sample_20-03-42.raw

D:\o31243\tSIM_data\20230614_SIM_TubeID_197_Sample_20-04-12.raw

D:\o31243\tSIM_data\20230614_SIM_TubeID_198_Sample_20-04-22.raw

D:\o31243\tSIM_data\20230614_SIM_TubeID_199_Sample_20-04-32.raw

D:\o31243\tSIM_data\20230614_SIM_TubeID_200_Sample_20-04-42.raw

D:\o31243\tSIM_data\20230614_SIM_TubeID_201_Sample_21-02-12.raw

D:\o31243\tSIM_data\20230614_SIM_TubeID_202_Sample_21-02-22.raw

D:\o31243\tSIM_data\20230614_SIM_TubeID_203_Sample_21-02-32.raw

D:\o31243\tSIM_data\20230614_SIM_TubeID_204_Sample_21-02-42.raw

D:\o31243\tSIM_data\20230614_SIM_TubeID_205_Sample_21-03-12.raw

D:\o31243\tSIM_data\20230614_SIM_TubeID_206_Sample_21-03-22.raw

D:\o31243\tSIM_data\20230614_SIM_TubeID_207_Sample_21-03-32.raw

D:\o31243\tSIM_data\20230614_SIM_TubeID_208_Sample_21-03-42.raw

D:\o31243\tSIM_data\20230614_SIM_TubeID_209_Sample_21-04-12.raw

D:\o31243\tSIM_data\20230614_SIM_TubeID_210_Sample_21-04-22.raw

D:\o31243\tSIM_data\20230614_SIM_TubeID_211_Sample_21-04-32.raw

D:\o31243\tSIM_data\20230614_SIM_TubeID_212_Sample_21-04-42.raw

D:\o31243\tSIM_data\20230614_SIM_TubeID_213_Sample_22-02-12.raw

D:\o31243\tSIM_data\20230614_SIM_TubeID_214_Sample_22-02-22.raw

D:\o31243\tSIM_data\20230614_SIM_TubeID_215_Sample_22-02-32.raw

D:\o31243\tSIM_data\20230614_SIM_TubeID_216_Sample_22-02-42.raw

D:\o31243\tSIM_data\20230614_SIM_TubeID_217_Sample_22-03-12.raw

D:\o31243\tSIM_data\20230614_SIM_TubeID_218_Sample_22-03-22.raw

D:\o31243\tSIM_data\20230614_SIM_TubeID_219_Sample_22-03-32.raw

D:\o31243\tSIM_data\20230614_SIM_TubeID_220_Sample_22-03-42.raw

D:\o31243\tSIM_data\20230614_SIM_TubeID_221_Sample_22-04-12.raw

D:\o31243\tSIM_data\20230614_SIM_TubeID_222_Sample_22-04-22.raw

D:\o31243\tSIM_data\20230614_SIM_TubeID_223_Sample_22-04-32.raw

D:\o31243\tSIM_data\20230614_SIM_TubeID_224_Sample_22-04-42.raw

D:\o31243\tSIM_data\20230614_SIM_TubeID_225_Sample_23-02-12.raw

D:\o31243\tSIM_data\20230614_SIM_TubeID_226_Sample_23-02-22.raw

D:\o31243\tSIM_data\20230614_SIM_TubeID_227_Sample_23-02-32.raw

D:\o31243\tSIM_data\20230614_SIM_TubeID_228_Sample_23-02-42.raw

D:\o31243\tSIM_data\20230614_SIM_TubeID_229_Sample_23-03-12.raw

D:\o31243\tSIM_data\20230614_SIM_TubeID_230_Sample_23-03-22.raw

D:\o31243\tSIM_data\20230614_SIM_TubeID_231_Sample_23-03-32.raw

D:\o31243\tSIM_data\20230614_SIM_TubeID_232_Sample_23-03-42.raw

D:\o31243\tSIM_data\20230614_SIM_TubeID_233_Sample_23-04-12.raw

D:\o31243\tSIM_data\20230614_SIM_TubeID_234_Sample_23-04-22.raw

D:\o31243\tSIM_data\20230614_SIM_TubeID_235_Sample_23-04-32.raw

D:\o31243\tSIM_data\20230614_SIM_TubeID_236_Sample_23-04-42.raw

D:\o31243\PRM_data\20230614_PRM_Blank2.raw

D:\o31243\PRM_data\20230614_PRM_Blank3.raw

D:\o31243\PRM_data\20230614_PRM_Blank4.raw

D:\o31243\PRM_data\20230614_PRM_Blank5.raw

D:\o31243\PRM_data\20230614_PRM_Blank6.raw

D:\o31243\PRM_data\20230614_PRM_Blank7.raw

D:\o31243\PRM_data\20230614_PRM_Blank8.raw

D:\o31243\PRM_data\20230614_PRM_Blank9.raw

D:\o31243\PRM_data\20230614_PRM_Blank10.raw

D:\o31243\PRM_data\20230614_PRM_QCpool_1.raw

D:\o31243\PRM_data\20230614_PRM_QCpool_2.raw

D:\o31243\PRM_data\20230614_PRM_QCpool_3.raw

D:\o31243\PRM_data\20230614_PRM_QCpool_4.raw

D:\o31243\PRM_data\20230614_PRM_QCpool_Steroids_mix_1fmol_1.raw

D:\o31243\PRM_data\20230614_PRM_QCpool_Steroids_mix_1fmol_3.raw

D:\o31243\PRM_data\20230614_PRM_QCpool_Steroids_mix_5fmol_1.raw

D:\o31243\PRM_data\20230614_PRM_QCpool_Steroids_mix_5fmol_3.raw

D:\o31243\PRM_data\20230614_PRM_QCpool_Steroids_mix_10fmol_1.raw

D:\o31243\PRM_data\20230614_PRM_QCpool_Steroids_mix_10fmol_3.raw

D:\o31243\PRM_data\20230614_PRM_QCpool_Steroids_mix_25fmol_1.raw

D:\o31243\PRM_data\20230614_PRM_QCpool_Steroids_mix_25fmol_3.raw

D:\o31243\PRM_data\20230614_PRM_QCpool_Steroids_mix_50fmol_1.raw

D:\o31243\PRM_data\20230614_PRM_QCpool_Steroids_mix_50fmol_3.raw

D:\o31243\PRM_data\20230614_PRM_QCpool_Steroids_mix_100fmol_1.raw

D:\o31243\PRM_data\20230614_PRM_QCpool_Steroids_mix_100fmol_3.raw

D:\o31243\PRM_data\20230614_PRM_QCpool_Steroids_mix_250fmol_1.raw

D:\o31243\PRM_data\20230614_PRM_QCpool_Steroids_mix_250fmol_3.raw

D:\o31243\PRM_data\20230614_PRM_Steroids_mix_1_25fmol_1.raw

D:\o31243\PRM_data\20230614_PRM_Steroids_mix_1_25fmol_2.raw

D:\o31243\PRM_data\20230614_PRM_Steroids_mix_1_25fmol_3.raw

D:\o31243\PRM_data\20230614_PRM_Steroids_mix_2_5fmol_1.raw

D:\o31243\PRM_data\20230614_PRM_Steroids_mix_2_5fmol_2.raw

D:\o31243\PRM_data\20230614_PRM_Steroids_mix_2_5fmol_3.raw

D:\o31243\PRM_data\20230614_PRM_Steroids_mix_5fmol_1.raw

D:\o31243\PRM_data\20230614_PRM_Steroids_mix_5fmol_2.raw

D:\o31243\PRM_data\20230614_PRM_Steroids_mix_5fmol_3.raw

D:\o31243\PRM_data\20230614_PRM_Steroids_mix_10fmol_1.raw

D:\o31243\PRM_data\20230614_PRM_Steroids_mix_10fmol_2.raw

D:\o31243\PRM_data\20230614_PRM_Steroids_mix_10fmol_3.raw

D:\o31243\PRM_data\20230614_PRM_Steroids_mix_20fmol_1.raw

D:\o31243\PRM_data\20230614_PRM_Steroids_mix_20fmol_2.raw

D:\o31243\PRM_data\20230614_PRM_Steroids_mix_20fmol_3.raw

D:\o31243\PRM_data\20230614_PRM_Steroids_mix_50fmol_1.raw

D:\o31243\PRM_data\20230614_PRM_Steroids_mix_50fmol_2.raw

D:\o31243\PRM_data\20230614_PRM_Steroids_mix_50fmol_3.raw

D:\o31243\PRM_data\20230614_PRM_Steroids_mix_100fmol_1.raw

D:\o31243\PRM_data\20230614_PRM_Steroids_mix_100fmol_2.raw

D:\o31243\PRM_data\20230614_PRM_Steroids_mix_100fmol_3.raw

D:\o31243\PRM_data\20230614_PRM_Steroids_mix_250fmol_1.raw

D:\o31243\PRM_data\20230614_PRM_Steroids_mix_250fmol_2.raw

D:\o31243\PRM_data\20230614_PRM_Steroids_mix_250fmol_3.raw

D:\o31243\PRM_data\20230614_PRM_Steroids_mix_500fmol_1.raw

D:\o31243\PRM_data\20230614_PRM_Steroids_mix_500fmol_2.raw

D:\o31243\PRM_data\20230614_PRM_Steroids_mix_500fmol_3.raw

D:\o31243\PRM_data\20230614_PRM_TubeID_2_Sample_01-02-22.raw

D:\o31243\PRM_data\20230614_PRM_TubeID_3_Sample_01-02-32.raw

D:\o31243\PRM_data\20230614_PRM_TubeID_4_Sample_01-02-42.raw

D:\o31243\PRM_data\20230614_PRM_TubeID_5_Sample_01-03-12.raw

D:\o31243\PRM_data\20230614_PRM_TubeID_6_Sample_01-03-22.raw

D:\o31243\PRM_data\20230614_PRM_TubeID_7_Sample_01-03-32.raw

D:\o31243\PRM_data\20230614_PRM_TubeID_8_Sample_01-03-42.raw

D:\o31243\PRM_data\20230614_PRM_TubeID_9_Sample_01-04-12.raw

D:\o31243\PRM_data\20230614_PRM_TubeID_10_Sample_01-04-22.raw

D:\o31243\PRM_data\20230614_PRM_TubeID_11_Sample_01-04-32.raw

D:\o31243\PRM_data\20230614_PRM_TubeID_12_Sample_01-04-42.raw

D:\o31243\PRM_data\20230614_PRM_TubeID_13_Sample_02-02-12.raw

D:\o31243\PRM_data\20230614_PRM_TubeID_14_Sample_02-02-22.raw

D:\o31243\PRM_data\20230614_PRM_TubeID_15_Sample_02-02-32.raw

D:\o31243\PRM_data\20230614_PRM_TubeID_16_Sample_02-02-42.raw

D:\o31243\PRM_data\20230614_PRM_TubeID_17_Sample_02-03-12.raw

D:\o31243\PRM_data\20230614_PRM_TubeID_18_Sample_02-03-22.raw

D:\o31243\PRM_data\20230614_PRM_TubeID_19_Sample_02-03-32.raw

D:\o31243\PRM_data\20230614_PRM_TubeID_20_Sample_02-03-42.raw

D:\o31243\PRM_data\20230614_PRM_TubeID_21_Sample_02-04-12.raw

D:\o31243\PRM_data\20230614_PRM_TubeID_22_Sample_02-04-22.raw

D:\o31243\PRM_data\20230614_PRM_TubeID_23_Sample_02-04-32.raw

D:\o31243\PRM_data\20230614_PRM_TubeID_24_Sample_02-04-42.raw

D:\o31243\PRM_data\20230614_PRM_TubeID_25_Sample_03-02-12.raw

D:\o31243\PRM_data\20230614_PRM_TubeID_26_Sample_03-02-22.raw

D:\o31243\PRM_data\20230614_PRM_TubeID_27_Sample_03-02-32.raw

D:\o31243\PRM_data\20230614_PRM_TubeID_28_Sample_03-02-42.raw

D:\o31243\PRM_data\20230614_PRM_TubeID_29_Sample_03-03-12.raw

D:\o31243\PRM_data\20230614_PRM_TubeID_30_Sample_03-03-22.raw

D:\o31243\PRM_data\20230614_PRM_TubeID_31_Sample_03-03-32.raw

D:\o31243\PRM_data\20230614_PRM_TubeID_32_Sample_03-03-42.raw

D:\o31243\PRM_data\20230614_PRM_TubeID_33_Sample_03-04-12.raw

D:\o31243\PRM_data\20230614_PRM_TubeID_34_Sample_03-04-22.raw

D:\o31243\PRM_data\20230614_PRM_TubeID_35_Sample_03-04-32.raw

D:\o31243\PRM_data\20230614_PRM_TubeID_36_Sample_03-04-42.raw

D:\o31243\PRM_data\20230614_PRM_TubeID_37_Sample_04-02-12.raw

D:\o31243\PRM_data\20230614_PRM_TubeID_38_Sample_04-02-22.raw

D:\o31243\PRM_data\20230614_PRM_TubeID_39_Sample_04-02-32.raw

D:\o31243\PRM_data\20230614_PRM_TubeID_40_Sample_04-02-42.raw

D:\o31243\PRM_data\20230614_PRM_TubeID_41_Sample_04-03-12.raw

D:\o31243\PRM_data\20230614_PRM_TubeID_42_Sample_04-03-22.raw

D:\o31243\PRM_data\20230614_PRM_TubeID_43_Sample_04-03-32.raw

D:\o31243\PRM_data\20230614_PRM_TubeID_44_Sample_04-03-42.raw

D:\o31243\PRM_data\20230614_PRM_TubeID_45_Sample_04-04-12.raw

D:\o31243\PRM_data\20230614_PRM_TubeID_46_Sample_04-04-22.raw

D:\o31243\PRM_data\20230614_PRM_TubeID_47_Sample_04-04-32.raw

D:\o31243\PRM_data\20230614_PRM_TubeID_48_Sample_04-04-42.raw

D:\o31243\PRM_data\20230614_PRM_TubeID_49_Sample_05-03-12.raw

D:\o31243\PRM_data\20230614_PRM_TubeID_50_Sample_05-03-22.raw

D:\o31243\PRM_data\20230614_PRM_TubeID_51_Sample_05-03-32.raw

D:\o31243\PRM_data\20230614_PRM_TubeID_52_Sample_05-03-42.raw

D:\o31243\PRM_data\20230614_PRM_TubeID_53_Sample_05-04-12.raw

D:\o31243\PRM_data\20230614_PRM_TubeID_54_Sample_05-04-22.raw

D:\o31243\PRM_data\20230614_PRM_TubeID_55_Sample_05-04-32.raw

D:\o31243\PRM_data\20230614_PRM_TubeID_56_Sample_05-04-42.raw

D:\o31243\PRM_data\20230614_PRM_TubeID_57_Sample_06-02-12.raw

D:\o31243\PRM_data\20230614_PRM_TubeID_58_Sample_06-02-22.raw

D:\o31243\PRM_data\20230614_PRM_TubeID_59_Sample_06-02-32.raw

D:\o31243\PRM_data\20230614_PRM_TubeID_60_Sample_06-02-42.raw

D:\o31243\PRM_data\20230614_PRM_TubeID_61_Sample_06-03-12.raw

D:\o31243\PRM_data\20230614_PRM_TubeID_62_Sample_06-03-22.raw

D:\o31243\PRM_data\20230614_PRM_TubeID_63_Sample_06-03-32.raw

D:\o31243\PRM_data\20230614_PRM_TubeID_64_Sample_06-03-42.raw

D:\o31243\PRM_data\20230614_PRM_TubeID_65_Sample_06-04-12.raw

D:\o31243\PRM_data\20230614_PRM_TubeID_66_Sample_06-04-22.raw

D:\o31243\PRM_data\20230614_PRM_TubeID_67_Sample_06-04-32.raw

D:\o31243\PRM_data\20230614_PRM_TubeID_68_Sample_06-04-42.raw

D:\o31243\PRM_data\20230614_PRM_TubeID_69_Sample_07-02-12.raw

D:\o31243\PRM_data\20230614_PRM_TubeID_70_Sample_07-02-22.raw

D:\o31243\PRM_data\20230614_PRM_TubeID_71_Sample_07-02-32.raw

D:\o31243\PRM_data\20230614_PRM_TubeID_72_Sample_07-02-42.raw

D:\o31243\PRM_data\20230614_PRM_TubeID_73_Sample_07-03-12.raw

D:\o31243\PRM_data\20230614_PRM_TubeID_74_Sample_07-03-22.raw

D:\o31243\PRM_data\20230614_PRM_TubeID_75_Sample_07-03-32.raw

D:\o31243\PRM_data\20230614_PRM_TubeID_76_Sample_07-03-42.raw

D:\o31243\PRM_data\20230614_PRM_TubeID_77_Sample_07-04-12.raw

D:\o31243\PRM_data\20230614_PRM_TubeID_78_Sample_07-04-22.raw

D:\o31243\PRM_data\20230614_PRM_TubeID_79_Sample_07-04-32.raw

D:\o31243\PRM_data\20230614_PRM_TubeID_80_Sample_07-04-42.raw

D:\o31243\PRM_data\20230614_PRM_TubeID_81_Sample_08-02-12.raw

D:\o31243\PRM_data\20230614_PRM_TubeID_82_Sample_08-02-22.raw

D:\o31243\PRM_data\20230614_PRM_TubeID_83_Sample_08-02-32.raw

D:\o31243\PRM_data\20230614_PRM_TubeID_84_Sample_08-02-42.raw

D:\o31243\PRM_data\20230614_PRM_TubeID_85_Sample_08-03-12.raw

D:\o31243\PRM_data\20230614_PRM_TubeID_86_Sample_08-03-22.raw

D:\o31243\PRM_data\20230614_PRM_TubeID_87_Sample_08-03-32.raw

D:\o31243\PRM_data\20230614_PRM_TubeID_88_Sample_08-03-42.raw

D:\o31243\PRM_data\20230614_PRM_TubeID_89_Sample_08-04-12.raw

D:\o31243\PRM_data\20230614_PRM_TubeID_90_Sample_08-04-22.raw

D:\o31243\PRM_data\20230614_PRM_TubeID_91_Sample_08-04-32.raw

D:\o31243\PRM_data\20230614_PRM_TubeID_92_Sample_08-04-42.raw

D:\o31243\PRM_data\20230614_PRM_TubeID_93_Sample_09-02-12.raw

D:\o31243\PRM_data\20230614_PRM_TubeID_94_Sample_09-02-22.raw

D:\o31243\PRM_data\20230614_PRM_TubeID_95_Sample_09-02-32.raw

D:\o31243\PRM_data\20230614_PRM_TubeID_96_Sample_09-02-42.raw

D:\o31243\PRM_data\20230614_PRM_TubeID_97_Sample_09-03-12.raw

D:\o31243\PRM_data\20230614_PRM_TubeID_98_Sample_09-03-22.raw

D:\o31243\PRM_data\20230614_PRM_TubeID_99_Sample_09-03-32.raw

D:\o31243\PRM_data\20230614_PRM_TubeID_100_Sample_09-03-42.raw

D:\o31243\PRM_data\20230614_PRM_TubeID_101_Sample_09-04-12.raw

D:\o31243\PRM_data\20230614_PRM_TubeID_102_Sample_09-04-22.raw

D:\o31243\PRM_data\20230614_PRM_TubeID_103_Sample_09-04-32.raw

D:\o31243\PRM_data\20230614_PRM_TubeID_104_Sample_09-04-42.raw

D:\o31243\PRM_data\20230614_PRM_TubeID_105_Sample_10-02-12.raw

D:\o31243\PRM_data\20230614_PRM_TubeID_106_Sample_10-02-22.raw

D:\o31243\PRM_data\20230614_PRM_TubeID_107_Sample_10-02-32.raw

D:\o31243\PRM_data\20230614_PRM_TubeID_108_Sample_10-02-42.raw

D:\o31243\PRM_data\20230614_PRM_TubeID_109_Sample_10-03-12.raw

D:\o31243\PRM_data\20230614_PRM_TubeID_110_Sample_10-03-22.raw

D:\o31243\PRM_data\20230614_PRM_TubeID_111_Sample_10-03-32.raw

D:\o31243\PRM_data\20230614_PRM_TubeID_112_Sample_10-03-42.raw

D:\o31243\PRM_data\20230614_PRM_TubeID_113_Sample_10-04-12.raw

D:\o31243\PRM_data\20230614_PRM_TubeID_114_Sample_10-04-22.raw

D:\o31243\PRM_data\20230614_PRM_TubeID_115_Sample_10-04-32.raw

D:\o31243\PRM_data\20230614_PRM_TubeID_116_Sample_10-04-42.raw

D:\o31243\PRM_data\20230614_PRM_TubeID_117_Sample_11-02-21.raw

D:\o31243\PRM_data\20230614_PRM_TubeID_118_Sample_11-02-22.raw

D:\o31243\PRM_data\20230614_PRM_TubeID_119_Sample_11-02-32.raw

D:\o31243\PRM_data\20230614_PRM_TubeID_120_Sample_11-02-42.raw

D:\o31243\PRM_data\20230614_PRM_TubeID_121_Sample_11-03-12.raw

D:\o31243\PRM_data\20230614_PRM_TubeID_122_Sample_11-03-22.raw

D:\o31243\PRM_data\20230614_PRM_TubeID_123_Sample_11-03-32.raw

D:\o31243\PRM_data\20230614_PRM_TubeID_124_Sample_11-03-42.raw

D:\o31243\PRM_data\20230614_PRM_TubeID_125_Sample_11-04-12.raw

D:\o31243\PRM_data\20230614_PRM_TubeID_126_Sample_11-04-22.raw

D:\o31243\PRM_data\20230614_PRM_TubeID_127_Sample_11-04-32.raw

D:\o31243\PRM_data\20230614_PRM_TubeID_128_Sample_11-04-42.raw

D:\o31243\PRM_data\20230614_PRM_TubeID_129_Sample_12-02-12.raw

D:\o31243\PRM_data\20230614_PRM_TubeID_130_Sample_12-02-22.raw

D:\o31243\PRM_data\20230614_PRM_TubeID_131_Sample_12-02-32.raw

D:\o31243\PRM_data\20230614_PRM_TubeID_132_Sample_12-02-42.raw

D:\o31243\PRM_data\20230614_PRM_TubeID_133_Sample_12-03-12.raw

D:\o31243\PRM_data\20230614_PRM_TubeID_134_Sample_12-03-22.raw

D:\o31243\PRM_data\20230614_PRM_TubeID_135_Sample_12-03-32.raw

D:\o31243\PRM_data\20230614_PRM_TubeID_136_Sample_12-03-42.raw

D:\o31243\PRM_data\20230614_PRM_TubeID_137_Sample_12-04-12.raw

D:\o31243\PRM_data\20230614_PRM_TubeID_138_Sample_12-04-22.raw

D:\o31243\PRM_data\20230614_PRM_TubeID_139_Sample_12-04-32.raw

D:\o31243\PRM_data\20230614_PRM_TubeID_140_Sample_12-04-42.raw

D:\o31243\PRM_data\20230614_PRM_TubeID_141_Sample_13-02-12.raw

D:\o31243\PRM_data\20230614_PRM_TubeID_142_Sample_13-02-22.raw

D:\o31243\PRM_data\20230614_PRM_TubeID_143_Sample_13-02-32.raw

D:\o31243\PRM_data\20230614_PRM_TubeID_144_Sample_13-02-42.raw

D:\o31243\PRM_data\20230614_PRM_TubeID_145_Sample_13-03-12.raw

D:\o31243\PRM_data\20230614_PRM_TubeID_146_Sample_13-03-22.raw

D:\o31243\PRM_data\20230614_PRM_TubeID_147_Sample_13-03-32.raw

D:\o31243\PRM_data\20230614_PRM_TubeID_148_Sample_13-03-42.raw

D:\o31243\PRM_data\20230614_PRM_TubeID_149_Sample_13-04-12.raw

D:\o31243\PRM_data\20230614_PRM_TubeID_150_Sample_13-04-22.raw

D:\o31243\PRM_data\20230614_PRM_TubeID_151_Sample_13-04-32.raw

D:\o31243\PRM_data\20230614_PRM_TubeID_152_Sample_13-04-42.raw

D:\o31243\PRM_data\20230614_PRM_TubeID_153_Sample_15-02-12.raw

D:\o31243\PRM_data\20230614_PRM_TubeID_154_Sample_15-02-22.raw

D:\o31243\PRM_data\20230614_PRM_TubeID_155_Sample_15-02-32.raw

D:\o31243\PRM_data\20230614_PRM_TubeID_156_Sample_15-02-42.raw

D:\o31243\PRM_data\20230614_PRM_TubeID_157_Sample_15-03-12.raw

D:\o31243\PRM_data\20230614_PRM_TubeID_158_Sample_15-03-22.raw

D:\o31243\PRM_data\20230614_PRM_TubeID_159_Sample_15-03-32.raw

D:\o31243\PRM_data\20230614_PRM_TubeID_160_Sample_15-03-42.raw

D:\o31243\PRM_data\20230614_PRM_TubeID_161_Sample_15-04-12.raw

D:\o31243\PRM_data\20230614_PRM_TubeID_162_Sample_15-04-22.raw

D:\o31243\PRM_data\20230614_PRM_TubeID_163_Sample_15-04-32.raw

D:\o31243\PRM_data\20230614_PRM_TubeID_164_Sample_15-04-42.raw

D:\o31243\PRM_data\20230614_PRM_TubeID_165_Sample_18-02-12.raw

D:\o31243\PRM_data\20230614_PRM_TubeID_166_Sample_18-02-22.raw

D:\o31243\PRM_data\20230614_PRM_TubeID_167_Sample_18-02-32.raw

D:\o31243\PRM_data\20230614_PRM_TubeID_168_Sample_18-02-42.raw

D:\o31243\PRM_data\20230614_PRM_TubeID_169_Sample_18-03-12.raw

D:\o31243\PRM_data\20230614_PRM_TubeID_170_Sample_18-03-22.raw

D:\o31243\PRM_data\20230614_PRM_TubeID_171_Sample_18-03-32.raw

D:\o31243\PRM_data\20230614_PRM_TubeID_172_Sample_18-03-42.raw

D:\o31243\PRM_data\20230614_PRM_TubeID_173_Sample_18-04-12.raw

D:\o31243\PRM_data\20230614_PRM_TubeID_174_Sample_18-04-22.raw

D:\o31243\PRM_data\20230614_PRM_TubeID_175_Sample_18-04-32.raw

D:\o31243\PRM_data\20230614_PRM_TubeID_176_Sample_18-04-42.raw

D:\o31243\PRM_data\20230614_PRM_TubeID_177_Sample_19-02-12.raw

D:\o31243\PRM_data\20230614_PRM_TubeID_178_Sample_19-02-22.raw

D:\o31243\PRM_data\20230614_PRM_TubeID_179_Sample_19-02-32.raw

D:\o31243\PRM_data\20230614_PRM_TubeID_180_Sample_19-02-42.raw

D:\o31243\PRM_data\20230614_PRM_TubeID_181_Sample_19-03-12.raw

D:\o31243\PRM_data\20230614_PRM_TubeID_182_Sample_19-03-22.raw

D:\o31243\PRM_data\20230614_PRM_TubeID_183_Sample_19-03-32.raw

D:\o31243\PRM_data\20230614_PRM_TubeID_184_Sample_19-03-42.raw

D:\o31243\PRM_data\20230614_PRM_TubeID_185_Sample_19-04-12.raw

D:\o31243\PRM_data\20230614_PRM_TubeID_186_Sample_19-04-22.raw

D:\o31243\PRM_data\20230614_PRM_TubeID_187_Sample_19-04-32.raw

D:\o31243\PRM_data\20230614_PRM_TubeID_188_Sample_19-04-42.raw

D:\o31243\PRM_data\20230614_PRM_TubeID_189_Sample_20-02-12.raw

D:\o31243\PRM_data\20230614_PRM_TubeID_190_Sample_20-02-22.raw

D:\o31243\PRM_data\20230614_PRM_TubeID_191_Sample_20-02-32.raw

D:\o31243\PRM_data\20230614_PRM_TubeID_192_Sample_20-02-42.raw

D:\o31243\PRM_data\20230614_PRM_TubeID_193_Sample_20-03-12.raw

D:\o31243\PRM_data\20230614_PRM_TubeID_194_Sample_20-03-22.raw

D:\o31243\PRM_data\20230614_PRM_TubeID_195_Sample_20-03-32.raw

D:\o31243\PRM_data\20230614_PRM_TubeID_196_Sample_20-03-42.raw

D:\o31243\PRM_data\20230614_PRM_TubeID_197_Sample_20-04-12.raw

D:\o31243\PRM_data\20230614_PRM_TubeID_198_Sample_20-04-22.raw

D:\o31243\PRM_data\20230614_PRM_TubeID_199_Sample_20-04-32.raw

D:\o31243\PRM_data\20230614_PRM_TubeID_200_Sample_20-04-42.raw

D:\o31243\PRM_data\20230614_PRM_TubeID_201_Sample_21-02-12.raw

D:\o31243\PRM_data\20230614_PRM_TubeID_202_Sample_21-02-22.raw

D:\o31243\PRM_data\20230614_PRM_TubeID_203_Sample_21-02-32.raw

D:\o31243\PRM_data\20230614_PRM_TubeID_204_Sample_21-02-42.raw

D:\o31243\PRM_data\20230614_PRM_TubeID_205_Sample_21-03-12.raw

D:\o31243\PRM_data\20230614_PRM_TubeID_206_Sample_21-03-22.raw

D:\o31243\PRM_data\20230614_PRM_TubeID_207_Sample_21-03-32.raw

D:\o31243\PRM_data\20230614_PRM_TubeID_208_Sample_21-03-42.raw

D:\o31243\PRM_data\20230614_PRM_TubeID_209_Sample_21-04-12.raw

D:\o31243\PRM_data\20230614_PRM_TubeID_210_Sample_21-04-22.raw

D:\o31243\PRM_data\20230614_PRM_TubeID_211_Sample_21-04-32.raw

D:\o31243\PRM_data\20230614_PRM_TubeID_212_Sample_21-04-42.raw

D:\o31243\PRM_data\20230614_PRM_TubeID_213_Sample_22-02-12.raw

D:\o31243\PRM_data\20230614_PRM_TubeID_214_Sample_22-02-22.raw

D:\o31243\PRM_data\20230614_PRM_TubeID_215_Sample_22-02-32.raw

D:\o31243\PRM_data\20230614_PRM_TubeID_216_Sample_22-02-42.raw

D:\o31243\PRM_data\20230614_PRM_TubeID_217_Sample_22-03-12.raw

D:\o31243\PRM_data\20230614_PRM_TubeID_218_Sample_22-03-22.raw

D:\o31243\PRM_data\20230614_PRM_TubeID_219_Sample_22-03-32.raw

D:\o31243\PRM_data\20230614_PRM_TubeID_220_Sample_22-03-42.raw

D:\o31243\PRM_data\20230614_PRM_TubeID_221_Sample_22-04-12.raw

D:\o31243\PRM_data\20230614_PRM_TubeID_222_Sample_22-04-22.raw

D:\o31243\PRM_data\20230614_PRM_TubeID_223_Sample_22-04-32.raw

D:\o31243\PRM_data\20230614_PRM_TubeID_224_Sample_22-04-42.raw

D:\o31243\PRM_data\20230614_PRM_TubeID_225_Sample_23-02-12.raw

D:\o31243\PRM_data\20230614_PRM_TubeID_226_Sample_23-02-22.raw

D:\o31243\PRM_data\20230614_PRM_TubeID_227_Sample_23-02-32.raw

D:\o31243\PRM_data\20230614_PRM_TubeID_228_Sample_23-02-42.raw

D:\o31243\PRM_data\20230614_PRM_TubeID_229_Sample_23-03-12.raw

D:\o31243\PRM_data\20230614_PRM_TubeID_230_Sample_23-03-22.raw

D:\o31243\PRM_data\20230614_PRM_TubeID_231_Sample_23-03-32.raw

D:\o31243\PRM_data\20230614_PRM_TubeID_232_Sample_23-03-42.raw

D:\o31243\PRM_data\20230614_PRM_TubeID_233_Sample_23-04-12.raw

D:\o31243\PRM_data\20230614_PRM_TubeID_234_Sample_23-04-22.raw

D:\o31243\PRM_data\20230614_PRM_TubeID_235_Sample_23-04-32.raw

D:\o31243\PRM_data\20230614_PRM_TubeID_236_Sample_23-04-42.raw

----

Processing node 33: Select Spectra

----

1. Spectrum Properties Filter:

- Lower RT Limit: 0

- Upper RT Limit: 0

- First Scan: 0

- Last Scan: 0

- Ignore Specified Scans: (not specified)

- Lowest Charge State: 0

- Highest Charge State: 0

- Min. Precursor Mass: 0 Da

- Max. Precursor Mass: 5000 Da

- Total Intensity Threshold: 0

- Minimum Peak Count: 1

2. Scan Event Filters:

- Mass Analyzer: (not specified)

- MS Order: Any

- Activation Type: (not specified)

- Min. Collision Energy: 0

- Max. Collision Energy: 1000

- Scan Type: Is Full

- Polarity Mode: (not specified)

- MS1 Mass Range: (not specified)

- FAIMS CV: (not specified)

3. Peak Filters:

- S/N Threshold (FT-only): 1.5

4. Replacements for Unrecognized Properties:

- Unrecognized Charge Replacements: 1

- Unrecognized Mass Analyzer Replacements: ITMS

- Unrecognized MS Order Replacements: MS2

- Unrecognized Activation Type Replacements: CID

- Unrecognized Polarity Replacements: +

- Unrecognized MS Resolution@200 Replacements: 60000

- Unrecognized MSn Resolution@200 Replacements: 30000

5. General Settings:

- Precursor Selection: Use MS(n - 1) Precursor

- Use Isotope Pattern in Precursor Reevaluation: True

- Provide Profile Spectra: Automatic

- Store Chromatograms: False

Processing node 46: Align Retention Times (ChromAlign)

----

1. General Settings:

- Reference File: 20230614_SIM_QCpool_2

Processing node 49: Detect Compounds

----

1. General Settings:

- Mass Tolerance [ppm]: 5 ppm

- Min. Peak Intensity: 10000

- Min. # Scans per Peak: 4

- Use Most Intense Isotope Only: True

2. Trace Detection:

- Max. Number of Gaps to Correct: 2

- Min. Number of Adjacent Non-Zeros: 2

3. Peak Detection:

- Chromatographic S/N Threshold: 1.5

- Remove Baseline: False

- Gap Ratio Threshold: 0.35

- Max. Peak Width [min]: 0.1

- Min. Relative Valley Depth: 0.1

4. Isotope Pattern Detection:

- Group Isotopes for: Br; Cl
- Use Peak Quality for Isotope Grouping: True
- Filter out Features with Bad Peaks Only: True
- Zig-Zag Index Threshold: 0.2
- Jaggedness Threshold: 0.4
- Modality Threshold: 0.9
- Remove Potentially False Positive Isotopes: True

5. Compound Detection:

- Ions:

    [2M+ACN+H]+1

    [2M+ACN+Na]+1

    [2M+FA-H]-1

    [2M+H]+1

    [2M+K]+1

    [2M+Na]+1

    [2M+NH4]+1

    [2M-H]-1

    [2M-H+HAc]-1

    [M+2H]+2

    [M+3H]+3

    [M+ACN+2H]+2

    [M+ACN+H]+1

    [M+ACN+Na]+1

    [M+Cl]-1

    [M+DMSO+H]+1

[M+FA-H]-1

[M+H]+1

[M+H+K]+2

[M+H+MeOH]+1

[M+H+Na]+2

[M+H+NH4]+2

[M+H-H2O]+1

[M+H-NH3]+1

[M+K]+1

[M+Na]+1

[M+NH4]+1

[M-2H]-2

[M-2H+K]-1

[M-H]-1

[M-H+HAc]-1

[M-H+TFA]-1

[M-H-H2O]-1

- Base Ions: [M+H]+1; [M-H]-1

- Remove Singlets: True

6. AcquireX Settings:

- Detect Persistent Background Ions: False

Processing node 31: Group Compounds

----

1. General Settings:

- Mass Tolerance: 5 ppm

- RT Tolerance [min]: 0.05

- Align Peaks: False
- Preferred Ions: [M+H]+1; [M-H]-1
- Area Integration: Most Common Ion

2. Peak Rating Contributions:
- Area Contribution: 3
- CV Contribution: 10
- FWHM to Base Contribution: 5
- Jaggedness Contribution: 5
- Modality Contribution: 5
- Zig-Zag Index Contribution: 5

3. Peak Rating Filter:
- Peak Rating Threshold: 5
- Number of Files: 1

Processing node 32: Fill Gaps

----

1. General Settings:
- Mass Tolerance: 5 ppm
- S/N Threshold: 1.5
- Use Real Peak Detection: True
- Apply Restrictive Gap Filling: True

Processing node 28: Mark Background Compounds

----

1. General Settings:

- Max. Sample/Blank: 5
- Max. Blank/Sample: 0
- Hide Background: True

Processing node 52: Normalize Areas

----

1. General Settings:

- Normalization Type: Constant Median
- Exclude Blanks: True

Processing node 25: Assign Compound Annotations

----

1. General Settings:

- Mass Tolerance: 5 ppm

2. Data Sources:

- Data Source #1: mzCloud Search
- Data Source #2: mzVault Search
- Data Source #3: MassList Search
- Data Source #4: ChemSpider Search
- Data Source #5: Predicted Compositions
- Data Source #6: (not specified)
- Data Source #7: (not specified)

3. Scoring Rules:

- Use mzLogic: True
- Use Spectral Distance: True

- SFit Threshold: 20

- SFit Range: 20

4. Reprocessing:

- Clear Names: False

Processing node 42: Search mzCloud

----

1. General Settings:

- Compound Classes: All

- Precursor Mass Tolerance: 10 ppm

- FT Fragment Mass Tolerance: 10 ppm

- IT Fragment Mass Tolerance: 0.4 Da

- Library: Autoprocessed; Reference

- Post Processing: Recalibrated

- Max. # Results: 10

- Annotate Matching Fragments: True

- Search MSn Tree: True

2. DDA Search:

- Identity Search: Cosine

- Match Activation Type: False

- Match Activation Energy: Any

- Activation Energy Tolerance: 20

- Apply Intensity Threshold: True

- Similarity Search: Confidence Forward

- Match Factor Threshold: 50

3. DIA Search:

- Use DIA Scans for Search: False

- Max. Isolation Width [Da]: 500

- Match Activation Type: False

- Match Activation Energy: Any

- Activation Energy Tolerance: 100

- Apply Intensity Threshold: False

- Match Factor Threshold: 20

Processing node 51: Search mzVault

----

1. Search Settings:

- mzVault Library: Bamba lab 34 lipid mediators library stepped NCE 10 30 45.db|Bamba lab 598 polar metabolites stepped NCE 10 30 45.db|LipidBlast-VS68-Neg.db|NIST_msms_metabolites.db|mzCloud Offline for mzVault_Endogenous_2021B.db|mzCloud_Offline for mzVault_Autoprocessed_2021B.db|mzCloud_Offline for mzVault_Endogenous-Autoprocessed_2021B.db|mzCloud_Offline for mzVault_Reference_2021B.db

- Max. # Results: 10

- Match Factor Threshold: 50

- Search Algorithm: NIST

- Match Analyzer Type: False

- IT Fragment Mass Tolerance: 0.4 Da

- FT Fragment Mass Tolerance: 10 ppm

- Use Retention Time: False

- Precursor Mass Tolerance: 10 ppm

- Apply Intensity Threshold: True

- Match Ionization Method: False

- Ion Activation Energy Tolerance: 20

- Match Ion Activation Energy: Match with Tolerance
- Match Ion Activation Type: False
- Compound Classes: All
- Remove Precursor Ion: True
- RT Tolerance [min]: 2

Processing node 40: Predict Compositions

----

1. Prediction Settings:

- Mass Tolerance: 5 ppm
- Min. Element Counts: C H
- Max. Element Counts: C90 H190 Br3 Cl4 N10 O18 P3 S5
- Min. RDBE: 0
- Max. RDBE: 40
- Min. H/C: 0.1
- Max. H/C: 3.5
- Max. # Candidates: 10
- Max. # Internal Candidates: 200

2. Pattern Matching:

- Intensity Tolerance [%]: 30
- Intensity Threshold [%]: 0.1
- S/N Threshold: 3
- Min. Spectral Fit [%]: 30
- Min. Pattern Cov. [%]: 90
- Use Dynamic Recalibration: True

3. Fragments Matching:

- Use Fragments Matching: True

- Mass Tolerance: 5 ppm

- S/N Threshold: 3

Processing node 41: Search ChemSpider

----

1. Search Settings:

- Database(s): BioCyc; Human Metabolome Database; KEGG

- Search Mode: By Formula or Mass

- Mass Tolerance: 5 ppm

- Max. # of results per compound: 100

- Max. # of Predicted Compositions to be searched per Compound: 3

- Result Order (for Max. # of results per compound): Order By Reference Count (DESC)

2. Predicted Composition Annotation:

- Check All Predicted Compositions: True

Processing node 48: Apply mzLogic

----

1. Search Settings:

- FT Fragment Mass Tolerance: 10 ppm

- IT Fragment Mass Tolerance: 0.4 Da

- Max. # Compounds: 0

- Max. # mzCloud Similarity Results to consider per Compound: 10

- Match Factor Threshold: 30

Processing node 50: Search Mass Lists

----

1. Search Settings:

- Mass Lists: Arita Lab 6549 Flavonoid Structure Database.masslist|Endogenous Metabolites database 4400 compounds.masslist|Natural Products Atlas 2021_08.masslist|Mass_List_Lipids_All_Splash_CD3_3.massList|IROA_WpH9_V2.massList

- Mass Tolerance: 5 ppm

- Use Retention Time: False

- RT Tolerance [min]: 0.8

Processing node 54: Descriptive Statistics

No parameters

Processing node 55: Differential Analysis

----

1. General Settings:

- Log10 Transform Values: True

2. Peak Rating Contributions:

- Update Peak Rating: True

- Area Contribution: 3

- CV Contribution: 10

- FWHM to Base Contribution: 5

- Jaggedness Contribution: 5

- Modality Contribution: 5

- Zig-Zag Index Contribution: 5

Filters to generate the curated table (83 entries)

This file contains the following filters:

Row Filter for Compounds:

```
-----------------------------------
AND
|
+--OR
|  |
|  +--mzVault Best Match is greater than 50.00
|  |
|  +--mzCloud Best Match is greater than 50.00
|
+--Annot. ΔMass [ppm] is between -5.00 and 5.00
|
+--Background is false
|
+--in category
   |
   +--Tags
   |
   +--2
-----------------------------------
```

Row Filter for WorkflowInputFile:

```
-----------------------------------
always true
```

Row Filter for StudyInformation:

----

always true

Row Filter for FileAlignmentResultItem:

----

always true

Row Filter for StatisticalReportItem:

----

always true

Row Filter for UnknownCompoundIonInstanceItem:

----

always true

Row Filter for UnknownCompoundInstanceItem:

----

always true

Row Filter for ChromatogramPeakItem:

----

always true

Row Filter for CompoundAnnotationProposalItem:

----

always true

Row Filter for MzCloudSearchResultItem:

----

always true

Row Filter for MzVaultSearchResultItem:

----

always true

Row Filter for PredictedCompositionItem:

----

always true

Row Filter for ChemSpiderResultItem:

----

always true

Row Filter for MassListSearchItem:

----

always true
