# Supplementary material for: The molecular signature of heat stress in sweat reveals non-invasive biomarker candidates for health monitoring
Source: Commun Biol. 2025 Apr 23;8:650. doi: 10.1038/s42003-025-08080-1 (PMC12019370; doi:10.1038/s42003-025-08080-1)
Supplement: Supplementary file 4 — Reporting Summary [file 42003_2025_8080_MOESM4_ESM.pdf]

nature portfolio

Corresponding author(s): Noé Brasier

Last updated by author(s): Apr 8, 2025

Reporting Summary

Nature Portfolio wishes to improve the reproducibility of the work that we publish. This form provides structure for consistency and transparency in reporting. For further information on Nature Portfolio policies, see our Editorial Policies and the Editorial Policy Checklist.

Statistics

For all statistical analyses, confirm that the following items are present in the figure legend, table legend, main text, or Methods section.

n/a	Confirmed	
☐	☒	The exact sample size (*n*) for each experimental group/condition, given as a discrete number and unit of measurement
☐	☒	A statement on whether measurements were taken from distinct samples or whether the same sample was measured repeatedly
☐	☒	The statistical test(s) used AND whether they are one- or two-sided<br>*Only common tests should be described solely by name; describe more complex techniques in the Methods section.*
☐	☒	A description of all covariates tested
☐	☒	A description of any assumptions or corrections, such as tests of normality and adjustment for multiple comparisons
☐	☒	A full description of the statistical parameters including central tendency (e.g. means) or other basic estimates (e.g. regression coefficient) AND variation (e.g. standard deviation) or associated estimates of uncertainty (e.g. confidence intervals)
☐	☒	For null hypothesis testing, the test statistic (e.g. *F*, *t*, *r*) with confidence intervals, effect sizes, degrees of freedom and *P* value noted<br>*Give P values as exact values whenever suitable.*
☒	☐	For Bayesian analysis, information on the choice of priors and Markov chain Monte Carlo settings
☐	☒	For hierarchical and complex designs, identification of the appropriate level for tests and full reporting of outcomes
☒	☐	Estimates of effect sizes (e.g. Cohen's *d*, Pearson's *r*), indicating how they were calculated

Our web collection on statistics for biologists contains articles on many of the points above.

Software and code

Policy information about availability of computer code

Data collection	RedCap (v12.4.17), iButton for real-life measurements (Thermodata Viewer v3.2.12), ECG Bittium (Bittium cardiac navigator software v1.5.6), BodyCap (monitor firmware v6.1.0 and ePerformance Manager v1.4.2)
Data analysis	The MS data generated with the metabolomic untargeted approach were processed by means of the commercial software Compound Discoverer 3.3 (Thermo Fisher Scientific), MS proteomics data processed for identification and quantification using the DIA-NN v1.8.1., Statistical analysis using R v4.3.2.

For manuscripts utilizing custom algorithms or software that are central to the research but not yet described in published literature, software must be made available to editors and reviewers. We strongly encourage code deposition in a community repository (e.g. GitHub). See the Nature Portfolio guidelines for submitting code & software for further information.

Data

Policy information about availability of data

All manuscripts must include a data availability statement. This statement should provide the following information, where applicable:

- Accession codes, unique identifiers, or web links for publicly available datasets
- A description of any restrictions on data availability
- For clinical datasets or third party data, please ensure that the statement adheres to our policy

Data availability statement is included in the manuscript.

Research involving human participants, their data, or biological material

Policy information about studies with human participants or human data. See also policy information about sex, gender (identity/presentation), and sexual orientation and race, ethnicity and racism.

Reporting on sex and gender	Participants were stratified for sex (10males, 10 females). Sex was self-reported by the participants upon enrollment. No subgroup analysis was conducted due to the small group sizes.
Reporting on race, ethnicity, or other socially relevant groupings	All participants underwent the same procedures. Participants were mostly recruited from higher educational institutions in Switzerland, thus representing a potential confounder.
Population characteristics	Healthy participants, able to give consent, Non-athlete (<4h sport/week), BMI<30 (non-obese), aged 18-40, German speaking, or fluent in German
Recruitment	Participants were recruited mostly through academic institutions. As most participants were students, this might had a potential impact for example in regard to physical fitness and daily heat exposure.
Ethics oversight	This observational study was approved by the Ethical Committee of Northwestern Switzerland (EKNZ ID 2022-01325).

Note that full information on the approval of the study protocol must also be provided in the manuscript.

Field-specific reporting

Please select the one below that is the best fit for your research. If you are not sure, read the appropriate sections before making your selection.

☒ Life sciences ☐ Behavioural & social sciences ☐ Ecological, evolutionary & environmental sciences

For a reference copy of the document with all sections, see nature.com/documents/nr-reporting-summary-flat.pdf

Life sciences study design

All studies must disclose on these points even when the disclosure is negative.

Sample size	20 assessed (23 included; 3 drop-outs)
Data exclusions	Due to sensor failure, two study visits had to be terminated early and thus were excluded from analysis. Three participants stopped their participation by themselves before an sweat samples were taken and without indicating reasons.
Replication	Findings were not replicated, the next clinical study will be set-up to extend and replicate the knowledge gained.
Randomization	The order of the study visits were assigned randomly. However, visits were not evenly distributed.
Blinding	No blinding.

Reporting for specific materials, systems and methods

We require information from authors about some types of materials, experimental systems and methods used in many studies. Here, indicate whether each material, system or method listed is relevant to your study. If you are not sure if a list item applies to your research, read the appropriate section before selecting a response.

Materials & experimental systems

n/a	Involved in the study	
☒	☐	Antibodies
☒	☐	Eukaryotic cell lines
☒	☐	Palaeontology and archaeology
☒	☐	Animals and other organisms
☐	☒	Clinical data
☒	☐	Dual use research of concern
☒	☐	Plants

Methods

n/a	Involved in the study	
☒	☐	ChIP-seq
☒	☐	Flow cytometry
☒	☐	MRI-based neuroimaging

Clinical data

Policy information about clinical studies

All manuscripts should comply with the ICMJE guidelines for publication of clinical research and a completed CONSORT checklist must be included with all submissions.

Clinical trial registration	ClinicalTrial.gov (NCT05622188)
Study protocol	The full study protocol was not published.
Data collection	A total of 23 participants were recruited between November 2022 and February 2023 (including 3 drop-outs). Data was collected at the Heat and Humidity Lab, Grenchen, Switzerland.
Outcomes	The study reported in this manuscript was part of and embedded into the Heatstar trial (please see NCT ID above). The outcomes for this part of the study were defined as the following: Novel Sweat Biomarkers for heat stress detection - Descriptive statistics for novel non-invasive biomarkers from sweat analysis with respect to heat stress source, including between- and within-subject variability.

Plants

Seed stocks	n/a
Novel plant genotypes	n/a
Authentication	n/a
